# CRISPR/Cas9 Genome Editing in *Caenorhabditis elegans*: Evaluation of Templates for Homology-Mediated Repair and Knock-Ins by Homology-Independent DNA Repair

**DOI:** 10.1534/g3.115.019273

**Published:** 2015-06-03

**Authors:** Iskra Katic, Lan Xu, Rafal Ciosk

**Affiliations:** Friedrich Miescher Institute for Biomedical Research, CH-4058 Basel, Switzerland

**Keywords:** genome editing, CRISPR, Cas9, NHEJ, *C. elegans*

## Abstract

Precise genome editing by the Cas9 nuclease depends on exogenously provided templates for homologous recombination. Here, we compare oligonucleotides with short homology and circular DNA molecules with extensive homology to genomic targets as templates for homology-based repair of CRISPR/Cas9 induced double-strand breaks. We find oligonucleotides to be templates of choice for introducing small sequence changes into the genome based on editing efficiency and ease of use. We show that polarity of oligonucleotide templates greatly affects repair efficiency: oligonucleotides in the sense orientation with respect to the target gene are better templates. In addition, combining a gene loss-of-function phenotype screen with detection of integrated fluorescent markers, we demonstrate that targeted knock-ins in *Caenorhabditis elegans* also can be achieved by homology-independent repair.

The repurposing of the *Streptococcus pyogenes* Cas9 nuclease (Jinek *et al.* 2012) has transformed genome editing in many organisms, including *C. elegans* [reviewed by Waaijers and Boxem (2014)]. This system consists, at its simplest, of an invariant Cas9 nuclease and a small chimeric guide RNA (sgRNA) containing 20 nucleotides of complementarity to the target DNA sequence (Jinek *et al.* 2012; 2013; Cong *et al.* 2013). The only absolute sequence requirement is the presence of the so-called protospacer adjacent motif (5′ NGG 3′) immediately downstream of the region of sgRNA complementarity in the genomic target (Jinek *et al.* 2012). Cas9-induced, double-stranded DNA breaks can be repaired either by nonhomologous end-joining (NHEJ) to introduce mutations in the genomic locus, or the repair can be templated by exogenous DNA, resulting in precise, homologous recombination-based, changes (Jinek *et al.* 2012; 2013; Cong *et al.* 2013; Mali *et al.* 2013).

Multiple approaches to obtaining *C. elegans* mutants by CRISPR/Cas9 have been published, yet they have not been methodically compared to maximize numbers of mutants obtained and minimize effort. We set out to compare different homologous recombination templates and resulting apparent recombinants, with the goal of being able to recommend preferred strategies.

We explored efficiency of homologous repair using commonly used templates—oligonucleotides with small regions of homology and circular DNA with extensive homology to the target site—because such a comparison has not yet been reported. Here we show that oligonucleotides are comparable with double-stranded plasmids as templates for homologous repair, but that there exists a polarity preference that affects their efficiency. We also show that single-stranded circular DNA can serve as a template for homologous repair after CRISPR/Cas9-induced double-stranded breaks. Finally, we demonstrate insertions of large constructs into precise sites in the *C. elegans* genome based on NHEJ.

## Materials and Methods

### Plasmids

The *PU6*::*unc-119* sgRNA plasmid (Friedland *et al.* 2013) was modified so that the *Not*I site was inserted between the K09B11.12 U6 promoter and the sgRNA backbone, deleting the *unc-119*-specific sgRNA sequences. This plasmid was termed pIK111. Gene-specific 20-nt sgRNA sequences were subsequently cloned in. First, an oligonucleotide of the form 5′ AATTGCAAATCTAAATGTTT(20 nt sgRNA-specific sequence) GTTTTAGAGCTAGAAATAGC 3′ was hybridized with its reverse and complementary oligonucleotide. The hybrid is then cloned into *Not*I-digested pIK111 by Gibson assembly (Gibson *et al.* 2009).

The *Peft-3*::*Cas9*::*tbb-2 3′UTR*, *PU6*::*sgRNA* plasmid pDD162 (Dickinson *et al.* 2013) was similarly modified to facilitate cloning of sgRNAs, if desired, resulting in plasmid pIK155.

The *PU6*::*sgRNA (F+E)* plasmid, pIK198 (#65629; Addgene), where the Cas9 binding region of the sgRNA was extended and a PolIII terminator removed (Chen *et al.* 2013a), was created by Gibson assembly of an IDT gBlock into a pUC57 plasmid digested with *Eco*RI. The same backbone was used by Ward (2015) in *C. elegans*; however, the U6 promoter used in that study (R07E5.16) differs from the one in *PU6*::*unc-119*, pIK111 and pIK198 (K09B11.12). Gene-specific, 20-nt sgRNA sequences subsequently were cloned in. An oligonucleotide of the form 5′ AATTGCAAATCTAAATGTTT (20-nt sgRNA-specific sequence) GTTTAAGAGCTATGCTGGAA 3′ was hybridized with its reverse and complementary oligonucleotide. The hybrid is cloned into *Not*I-digested pIK198 by Gibson assembly (Gibson *et al.* 2009).

The nonhomologous end joining templates for *unc-22* and *lin-41* were created by inserting hybridized oligonucleotides containing the gene-specific sgRNA, 4 bp upstream and 6 bp downstream from its endogenous genomic locus and 20 bp homology arms, into the *Eco*RI site of pIK127 (*Peft-3*::*gfp*::*h2b*::*tbb-2 3′UTR*) (#65631; Addgene) or *Bgl*II site of pIK137 (*Peft-3*::*gfp*::*h2b*::*tbb-2 3′UTR*, *C. briggsae unc-119*) (#65632; Addgene) by Gibson assembly.

Phagemid templates for *sqt-1* and *lin-12* experiments were created by Gibson assembly of polymerase chain reaction (PCR) products from recombinant animals from oligonucleotide-templated experiments into pBluescript SK+. The *sqt-1* phagemid has homology arms of 1.3 and 1.7 kb, respectively. The *lin-12* phagemid has homology arms of 1.5 kb each.

### Oligonucleotides

**Figure 1:**
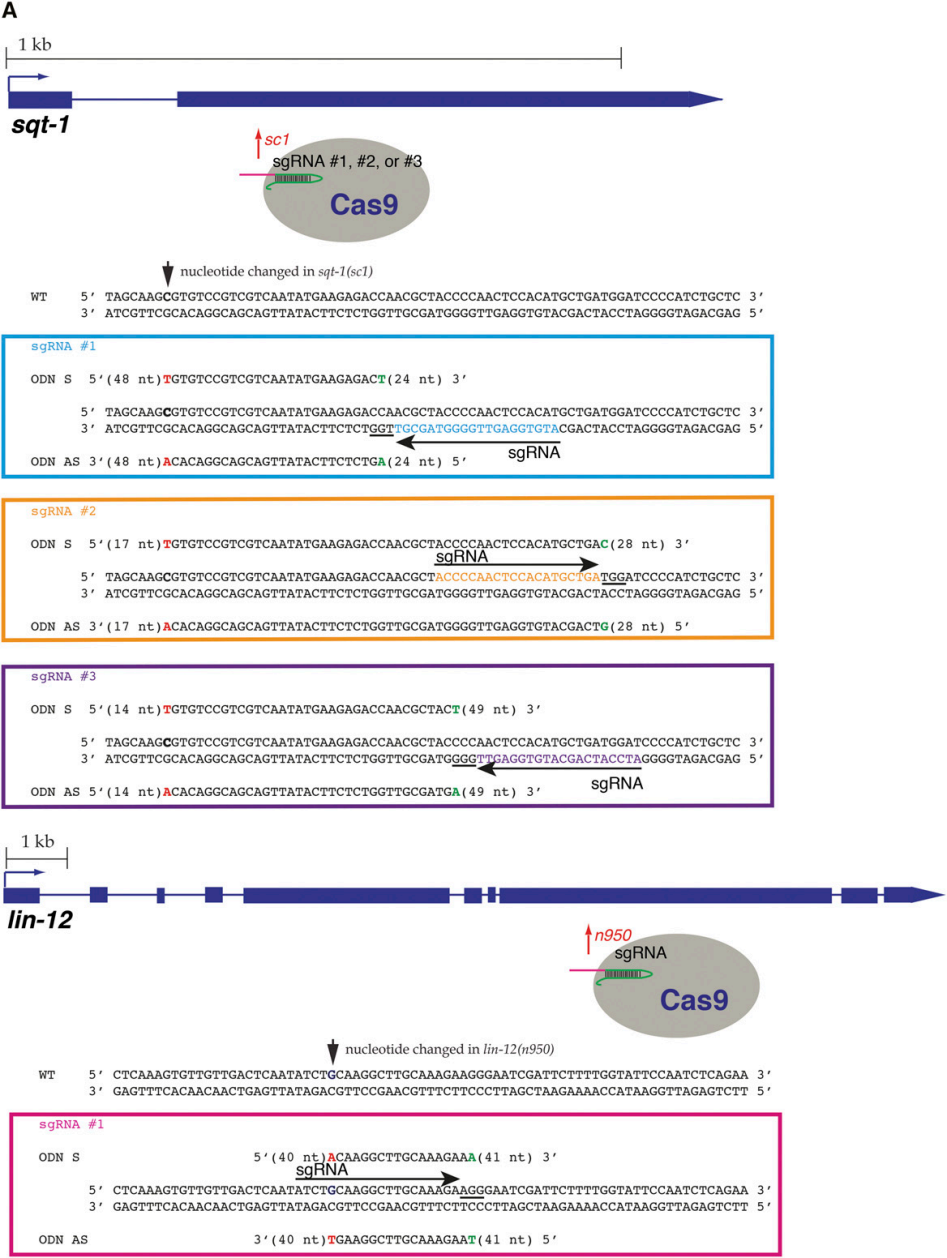

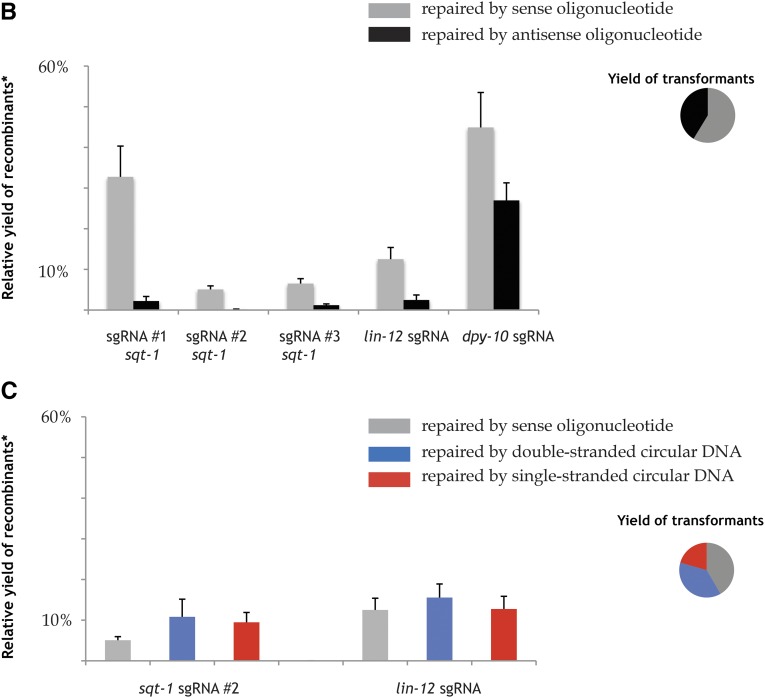
Homologous repair frequencies upon CRISPR/Cas9-induced double-strand breaks through oligonucleotides, double- and single-stranded circular DNA. (A) Sequences of three sgRNAs targeting *sqt-1* and one sgRNA targeting *lin-12*, and DNA oligonucleotides (ODN) used as homologous recombination templates. 20-nt sgRNA target binding sites are shown as arrows next to the strand each is homologous to. Protospacer adjacent motif nucleotides are underlined. The nucleotide whose change results in a dominant mutation is shown in bold on the coding strand of the gene (C in *sqt-1*, G in *lin-12*). All oligonucleotides are 99-mers and contain the causative mutation, in red (T in *sqt-1*, A in *lin-12*) and a silent mutation, in green. For each oligonucleotide, the graphic shows the sequence of the two mutations and nucleotides between them, and gives the number of nucleotides in either recombination arm of the oligonucleotide. (B) Efficiency of repair by DNA oligonucleotides in the sense (gray) or antisense directions (black) in experiments using Cas9 and each sgRNA in turn. *Relative yield of recombinants in each experiment is calculated by dividing the number of mutant F_1_s with heritable mutations from each experiment by the number of green fluorescent protein (GFP)-positive animals resulting from the experiment, as a measure of microinjection efficiency. Error bars represent SEM (n = 3 separate experiments for each category). The pie chart represents the yield of GFP positive animals in 15 experiments using sense and 15 experiments using antisense oligonucleotides, respectively (1824 *vs.* 1283 animals). (C) Efficiency of repair by DNA oligonucleotides in the sense direction (gray), double-stranded (blue), and single-stranded circular DNA (red) in experiments using Cas9 and one sgRNA targeting *sqt-1* and *lin-12*, respectively. *Relative yield of recombinants in each experiment is calculated by dividing the number of mutant F_1_s with heritable mutations from each experiment by the number of GFP-positive animals resulting from experiment, as a measure of micoinjection efficiency. Error bars represent SEM (n = 3 separate experiments for each category). The ± 95% CI intervals were 5.0 ± 3.4, 10.8 ± 17.0, and 9.5 ± 9.5% for *sqt-1* sgRNA #2 oligonucleotides, double-stranded and single-stranded circular DNA templates, and 12.5 ± 11.3, 15.6 ± 13.1, and 12.7 ± 12.2% for *lin-12* sgRNA oligonucleotides, double-stranded and single-stranded circular DNA templates, respectively. The pie chart represents the yield of GFP-positive animals in 6 experiments using sense oligonucleotides (784 animals), 6 experiments using double-stranded DNA (713 animals), and 6 experiments using single-stranded DNA (387 animals).

All oligonucleotide homologous recombination templates were ordered from (Integrated DNA Technologies) IDT and purified by polyacrylamide gel electrophoresis. Sequences for homologous recombination template oligonucleotides for *sqt-1* and *lin-12* modification are presented in Figure 1A. The sense repair oligonucleotide for *dpy-10(cn64)* was AF-ZF-827 (Arribere *et al.* 2014); the antisense oligo was its reverse and complement, oIK770.

### Injection mixes

All plasmids used for injection were purified by Nucleobond Xtra midi and Finalizer plus (740410.50 and 740520.20; MACHEREY-NAGEL). For *sqt-1(sc1)*, *lin-12(n950)*, and *dpy-10(cn64)* phenocopy, injection mixes contained 50 ng/μL pIK155 *Peft-3*::*Cas9*::*tbb-2* 3′ UTR, 100 ng/μL gene-specific sgRNA construct in pIK111 (*sqt-1*, *lin-12*) or pIK198 (*dpy-10*) *PU6*::*sgRNA* plasmid, 20 ng/μL *Pmyo-3*::*gfp*, and 50 ng/μL template oligonucleotide, or 100 ng/μL double-stranded circular template, or 50 ng/μL single-stranded circular template. Injections were performed in N2 animals. For comparison with the R07E5.16 promoter, *sqt-1(sc1)* sgRNA #2 was cloned in pIK155 *Peft-3*::*Cas9*::*tbb-2 3′UTR*, *PU6*::*sgRNA* plasmid. The injection mix contained 50 ng/μL of the resulting plasmid pIK177, 50 ng/μL template oligonucleotide, and 20 ng/μL *Pmyo-3*::*gfp*.

For *unc-22* mutagenesis, injection mixes contained 50 ng/μL pIK155 *Peft-3*::*Cas9*::*tbb-2* 3′ UTR, 100 ng/μL gene-specific sgRNA construct in pIK198 *PU6*::*sgRNA* plasmid, and 20 ng/μL *Pmyo-3*::*gfp*. Injections were performed in N2 animals.

For inserting *Peft-3*::*gfp*::*h2b*::*tbb-2 3′UTR* into *unc-22*, the mix contained 5 ng/μL pCFJ104, 5 ng/μL pGH8, 2.5 ng/μL pCFJ90 (Frøkjær-Jensen *et al.* 2012), 100 ng/μL pIK155 *Peft-3*::*Cas9*::*tbb-2* 3′ UTR, 100 ng/μL pIK199 (5′ GCTCCATTGGTATGGTACCG 3′ sgRNA in F+E backbone pIK198 plasmid), 100 ng/μL pIK206 (5′ GACAAGCCGAAACCACCAAA 3′ sgRNA in F+E backbone pIK198 plasmid), and 100 ng/μL pIK211 (5′ TAAGGACAAGCCGAAACCACCAAAGGGTCC 3′ cloned in pIK137 [*Peft-3*::*gfp*::*h2b*::*tbb-2 3′UTR*, *C. briggsae unc-119*]). The mix was injected into HT1593 *unc-119(ed3)* animals.

For inserting *Peft-3*::*gfp*::*h2b*::*tbb-2 3′UTR* into *lin-41*, the mix contained 5 ng/μL pCFJ104, 5 ng/μL pGF8, 2.5 ng/μL pCFJ90 (Frøkjær-Jensen *et al.* 2012), 100 ng/μL pIK155 *Peft-3*::*Cas9*::*tbb-2* 3′ UTR, 100 ng/μL pIK123 (5′ GCTTCAAACTGAGATCGACG 3′ sgRNA in backbone pIK111 plasmid), and 100 ng/μL pIK204 (5′ GTCGGCTTCAAACTGAGATCGACGTGGACG 3′ cloned in pIK127 [*Peft-3*::*gfp*::*h2b*::*tbb-2 3′UTR*]). The mix was injected into N2 animals. In both cases, F_2_ progeny were screened for individuals exhibiting ubiquitous green fluorescence and no red fluorescence.

### Single-stranded circular DNA generation

The double-stranded DNA in pBluescript SK+ phagemid vector was transformed into an F´ containing an *Escherichia coli* strain (XL1 Blue; Agilent Technologies). Transformants were infected with M13K07 helper phage as described in the manufacturer’s protocol (New England Biolabs). After harvesting cells by centrifugation, single-stranded DNA was purified by the PureLink HiPure Plasmid midiprep kit (Invitrogen; protocol version for single-stranded DNA purification).

### PCR identification of NHEJ-mediated insertions in *unc-22* and *lin-41*

The *unc-22(bch26)* insertion was identified by the following PCRs:

oIK803 (5′-AGAGAAGACCGTTCAACAACAGG-3′, in the *unc-22* locus) –oIK777 (5′- TGCACATCTAACTCCTAGCACG-3′, in *Peft-3* on the repair plasmid) and
oIK808 (5′-AGCCTCGTTCATCTCGATCTTTC-3′, in the *unc-22* locus) – oIK818 (5′- TATGCGGCATCAGAGCAGATTG-3′, in the repair plasmid backbone).
oIK807 (5′-AGGAGCCACGGCATACATTC-3′, in the *unc-22* locus) - oIK810 (5′-TGCCAATCTTTCTCGGACTTCTC-3′, in the *unc-22* locus) PCR was performed to confirm that the *unc-22* genomic locus was not modified through a sgRNA #1-mediated cut.

The *lin-41(bch28)* insertion was identified by the following PCR:

oIK796 (5′-CTTGTCATCAGCAGCCCTCG-3′, in the *lin-41* locus) – oIK777 (5′- TGCACATCTAACTCCTAGCACG-3′, in *Peft-3* on the repair plasmid)
oIK817 (5′- GTCCCGAGCCAAGATGATTATCC-3′, in the *lin-41* locus) – oIK819 (5′- CTGCACATCTAACTCCTAGCACG-3′, in *Peft-3* on the repair plasmid)

## Results and Discussion

### Oligonucleotides in the sense orientation with respect to the target gene are better repair templates

Repair of CRISPR/Cas9-induced lesions by oligonucleotides has been reported in *C. elegans* (Arribere *et al.* 2014; Zhao *et al.* 2014; Ward 2015). To compare relative repair frequencies by different templates, we focused on phenocopying three dominant mutations, *sqt-1(sc1)* (Cox *et al.* 1980), *lin-12(n950)* (Greenwald *et al.* 1983), and *dpy-10(cn64)* (Arribere *et al.* 2014), as phenotypic screening for recombinants can be performed in the first filial generation: *lin-12(n950)/+* animals have a Multivulva phenotype, whereas *sqt-1(sc1)/+* and *dpy-10(cn64)/+* animals roll. We designed three sgRNAs to target *sqt-1*, one to target *lin-12* (Figure 1A), and used a previously published sgRNA targeting *dpy-10* (Arribere *et al.* 2014). In each experiment, wild-type (N2) animals were microinjected with a mix containing ubiquitously expressed Cas9, an sgRNA, a repair oligonucleotide in the sense or antisense direction with respect to the transcription of the gene, and a fluorescent marker. The oligonucleotides encode desired changes, which result in dominant phenotypes, and a silent change. Some of the silent changes disrupt sgRNA binding and prevent recutting of the recombinant genomic locus by Cas9. Each experiment was repeated three times, by two independent experimenters, and apparent F_1_ recombinants—roller animals or animals with multivulva phenotype, respectively—were scored for heritable phenotype segregation and presence of fluorescent marker expression (Table 1). The yield of recombinants resulting from each injection was normalized to the number of transgenic animals resulting from the injection; this normalization accounts for the efficiency of the microinjection procedure and any toxicity of the DNA mix.

**Table 1:** Heritability of dominant mutant phenotypes in F1 animals and their cosegregation with a fluorescent transformation marker

sgRNA and Recombination Template	Number of Mutant F_1_s	Heritable (%)	Of Heritable, Fluorescent (%)
Oligonucleotide templates			
* sqt-1* sgRNA #1 sense	68	62 (91)	2 (4)*^a^*
* sqt-1* sgRNA #1 antisense	2	2 (100)	0
* sqt-1* sgRNA #2 sense	25	19 (76)	0
* sqt-1* sgRNA #2 antisense	3	1 (33)	0
* sqt-1* sgRNA #3 sense	36	31 (86)	4 (13)
* sqt-1* sgRNA #3 antisense	16	7 (44)	1 (14)
* lin-12* sense	125	42 (34)	12 (29)
* lin-12* antisense	73	4 (5)	3 (75)
* dpy-10* sense	273	143 (52)	53 (37)
* dpy-10* antisense	198	118 (60)	36 (31)
Total oligonucleotide	819	429 (52)	111 (27)*^a^*
Double-stranded circular templates			
* sqt-1* sgRNA #2	99	9 (9)	0
* lin-12*	125	35 (28)	14 (40)
Total double-stranded DNA	224	44 (20)	14 (32)
Single-stranded circular templates			
* sqt-1* sgRNA #2	105	15 (14)	0
* lin-12*	118	27 (23)	9 (33)
Total single-stranded DNA	223	42 (19)	9 (21)

The table shows the total number of mutant (roller or multivulva) animals resulting from microinjections with three sgRNAs targeting *sqt-1*, one sgRNA targeting *lin-12*, one sgRNA targeting *dpy-10*, and the Cas9 driven by the ubiquitous promoter of the *eft-3* gene. Each mix was injected into 20-30 P_0_ animals in triplicate, except for *dpy-10* sgRNA containing mixes, which were injected into 10-15 P_0_ animals in triplicate. Heritable changes are those that mutant animals segregate in the F_2_ generation (roller, dumpy, multivulva, and egg-laying deficient, respectively). The last column shows the proportion of F_1_ animals with heritable mutations which were also positive for the transgenic array.

The “Total oligonucleotide” row includes results of 30 separate microinjections with sense or antisense oligonucleotides as recombination templates.

a Fluorescence status was known for just 412 animals. The “Total double-stranded DNA” and “Total single-stranded DNA” rows include results of 6 separate injections each.

In each case, we found that the oligonucleotide sense to the direction of gene transcription was a better recombination template, regardless of the DNA strand the sgRNA was complementary to (Figure 1B); observations consistent with ours also were made by Ward (2015) from a small number of co-conversion experiments. From our two studies, we have evidence that oligonucleotides sense to the direction of gene transcription are better recombination templates for seven pairs of oligonucleotides, targeting five genes, and irrespective of the DNA strand the sgRNA targets (Supporting Information, Table S1).

Similarly to Zhao *et al.* (2014) and Arribere *et al.* (2014), we find that recombinants are preferentially nontransgenic, *i.e.*, they do not inherit the DNA array containing fluorescent markers, Cas9, sgRNAs, and oligonucleotide templates (Table 1), which has implications for experimental design. We also find that, using the Cas9 protein expressed from a ubiquitous *eft-3* promoter, we obtain a proportion of apparent recombinant animals (rollers and animals with multivulvae, respectively) segregating only wild-type progeny (Table 1); it is likely that in these animals, recombination occurs in one or more somatic tissues during development.

In all experiments described here, we express sgRNAs from the K09B11.12 U6 promoter (Friedland *et al.* 2013). Farboud and Meyer (2015) reported failure to obtain *rol-6(su1006)* mutants using the K09B11.12 promoter, but were successful using the R07E5.16 promoter originally described by Dickinson *et al.* (2013). To compare these two promoters, we scored yields of heritable roller animals obtained from driving *sqt-1(sc1)* sgRNA #2 expression from the R07E5.16 promoter in the pDD162 Cas9 plasmid (Dickinson *et al.* 2013) with those obtained using the K09B11.12 promoter, in the context of the gene conversion mix with the oligonucleotide sense to *sqt-1* transcription. The 95% confidence interval using 100 ng/μl of the K09B11.12-driven sgRNA was 5.0 ± 3.4% recombinants; for 50 ng/μL of the R07E5.16 promoter-driven sgRNA, it was 0.5 ± 0.1% recombinants. Thus, we continued to use the K09B11.12 promoter, which is also effective in co-conversion experiments (I. Katic, unpublished data).

### Single-stranded oligonucleotides and double-stranded or single-stranded circular DNA with extensive homology to the target locus are comparable templates for homologous recombination (HR)

It had been reported that single-stranded DNA might be a better template than double-stranded DNA in fission yeast (Simon and Moore 1987) and that it can act as a HR template in mammalian cells (Fujioka *et al.* 1993). We tested whether this might be the case in *C. elegans*.

We again assayed phenocopy of dominant mutations in *sqt-1* and *lin-12*, now using double-stranded phagemid DNA with >1 kb homology arms and corresponding circular single-stranded DNAs. We do not observe significant differences between sense oligonucleotides, double- and single-stranded circular DNA (Figure 1C), so for ease of experimental design, we recommend oligonucleotides as templates for nucleotide changes and short tag insertions. With all kinds of templates, we found that recombinants were found more often among nontransgenic animals: more than 70% of all recombination events happened in animals that did not inherit the fluorescent array (Table 1). If HR events are desired, therefore, co-conversion approaches (Arribere *et al.* 2014; Ward 2015) where an oligonucleotide-templated HR event at one locus is used to enrich for a desired modification of a different locus, appear to be preferable to cloning transgenic F_1_ animals and analyzing their progeny. We also attempted to generate single-stranded linear DNA templates by restriction digest of single-stranded circular DNA, but we observed widespread toxicity (I. Katic, unpublished data).

### sgRNAs 2ith target site homology of >20 nt can also guide Cas9

We and others have previously shown that mismatches at the 5′ end of an sgRNA can be tolerated (Jinek *et al.* 2012; Cong *et al.* 2013; Fu *et al.* 2013; Hwang *et al.* 2013; Katic and Großhans 2013; Paix *et al.* 2014; Ward 2015; Farboud and Meyer 2015). DNA-based CRISPR approaches in *C. elegans* use U6 promoters to express sgRNAs (Friedland *et al.* 2013; Dickinson *et al.* 2013; Chen *et al.* 2013b). Although the exact sequence requirements of *C. elegans* U6 promoters have not been studied, transcription from a mouse U6 promoter initiates at the first A or G nucleotide starting from the annotated −1 position (Ma *et al.* 2014). The apparent requirement of a starting A or G reduces the choices of available sgRNAs with perfect complementarity to the target.

We compared a series of sgRNAs targeting the same sequence within *sqt-1* but differing in length. All three sgRNAs are perfectly complementary to the target sequence and all have an A as the 5′-most nucleotide. They are 20, 26, and 30 nucleotides long. Although microinjection of the 20-nt sgRNA results in the greatest proportion of recombinants with respect to transgenic animals obtained (32%), the remaining sgRNAs also can form functional complexes with Cas9 (13% and 4% recombinants, respectively; Table S2), as was shown in mammalian systems (Qi *et al.* 2013; Ran *et al.* 2013). This flexibility in choosing an sgRNA of different length, in addition to the possibility of adding 5′ A or G nucleotides noncomplementary to the template (Katic and Großhans 2013; Paix *et al.* 2014; Ward 2015; Farboud and Meyer 2015) increases the pool of sgRNAs available to the *C. elegans* experimenter.

### sgRNA effects on mutation rate

Efficiency of Cas9-mediated genome engineering crucially depends on sgRNA efficacy, which varies widely (summarized by Farboud and Meyer (2015) for *C. elegans*). To improve the stability of the sgRNA backbone, we synthesized a “flipped and extended” version (Chen *et al.* 2013a), where an A-U basepair is flipped within the sgRNA stem-loop to disrupt a potential Pol III terminator and the stem-loop is extended by 5 bp. We placed this new backbone behind the K09B11.12 U6 promoter (Friedland *et al.* 2013) to create a universal sgRNA cloning vector, pIK198, which will be available through Addgene. The identical backbone was used by Ward (2015), but the snRNA promoter in that study, R07E5.16, is different.

To test a model for prediction of sgRNA activity based on experimental data in mammalian systems (Doench *et al.* 2014), we designed four sgRNAs targeting the long 20th exon of the *unc-22* gene and expressed them from the improved backbone. Two were predicted to be efficient (score >0.7) and two were poor (score <0.1). We tested the mutagenicity of each sgRNA separately and found that the two sgRNAs predicted to be efficient indeed resulted in more Unc-22 animals in total than the two predicted to be inefficient (102 *vs.* 15 mutant animals obtained; Table S3). In addition, we analyzed *C. elegans* sgRNAs with published mutagenesis efficiencies according to the Doench *et al.* (2014) algorithm. Of the 46 sgRNAs that met our criteria, 34 showed a range of levels of activity, while 12 did not result in any mutants. Of the 12 inactive sgRNAs, 10 have scores of <0.2 in the Doench *et al.* (2014) algorithm, one has a score of 0.25, and one a high score of 0.67 (Table S4). Furthermore, of the 23 sgRNAs with scores of <0.2, 10 are inactive. Recently, Farboud and Meyer (2015) proposed and validated a model for designing efficient sgRNAs targeting *C. elegans* genes whose requirements are 5′ N_17_NGG(NGG)3′, where the protospacer adjacent motif sequence is in parentheses. When these sequence requirements are difficult to meet for the desired modification, our analysis suggest that low scoring sgRNAs from the Doench *et al.* (2014) model should be used with caution, and high scoring ones considered.

### NHEJ-mediated knock-ins

Precise genome modification by homologous recombination is crucial in many cases, such as when precisely tagging a protein. However, despite continued improvements in screening techniques (Kim *et al.* 2014; Arribere *et al.* 2014; Ward 2015) and template optimization (Paix *et al.* 2014), we and others have had difficulties achieving consistent HR-mediated modification events (Farboud and Meyer (2015), and our unpublished results). On the other hand, NHEJ is clearly active in the germline of *C. elegans*, or in very early embryos, as heritable mutations resulting from Cas9 when a recombination template is not provided are repaired by that mechanism. NHEJ approaches have been used to achieve knock-ins upon ZFN and TALEN cleavage in cell lines (Maresca *et al.* 2013), as well as Cas9-mediated cleavage in zebrafish (Auer *et al.* 2014).

**Figure 2:**
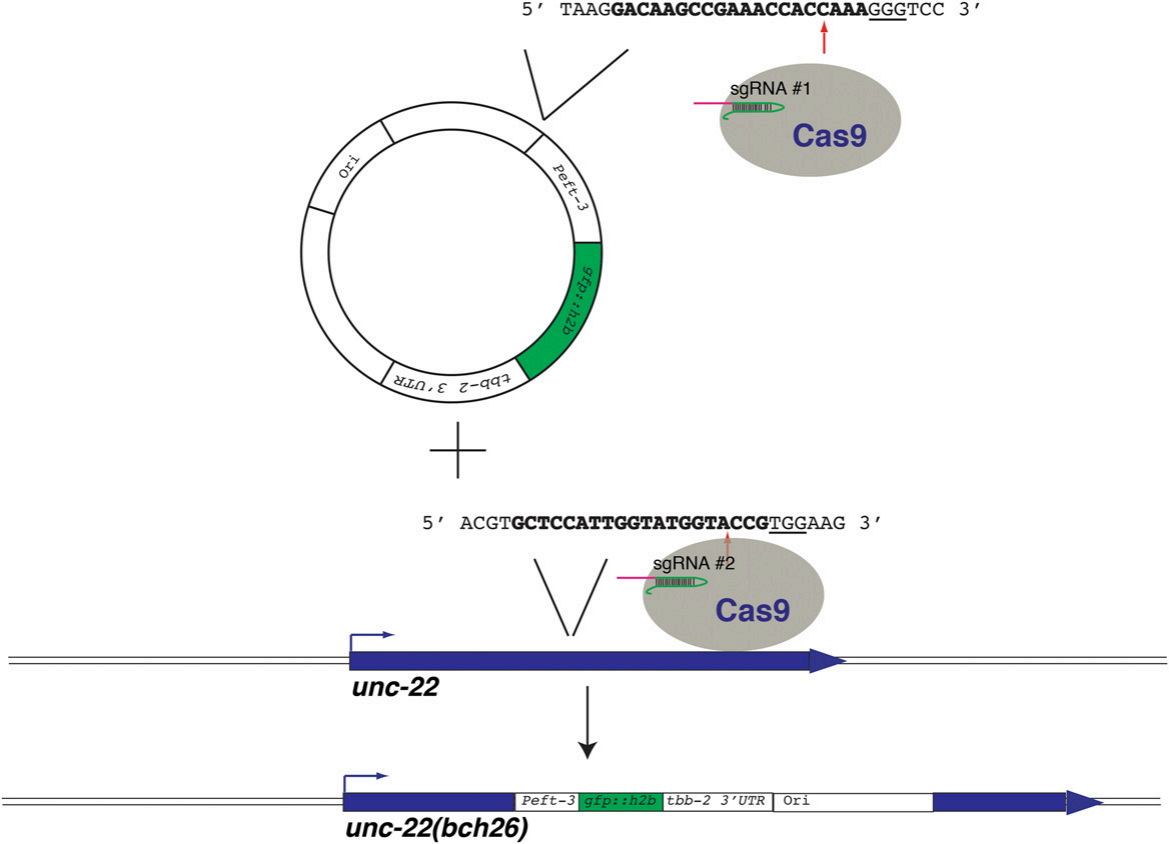
Knock-in of a plasmid into a genomic locus upon nonhomologous end joining-mediated repair of a Cas9/CRISPR lesion. Knock-in of a *Peft-3*::*gfp*::*h2b*::*tbb-2 3′UTR* –containing plasmid into the *unc-22* locus. The 30-bp genomic sequence from the *unc-22* locus was cloned into the *Peft-3*::*gfp*::*h2b*::*tbb-2 3′UTR*, *C. briggsae unc-119* rescue fragment –containing plasmid, including the 20-bp sgRNA #1 target site (bold) and the protospacer adjacent motif sequence (underlined). Cas9 guided by two *unc-22* sgRNAs concomitantly cuts the plasmid and the genomic locus, which can then lead to insertion of the cut plasmid into the genomic lesion. In the case of the insertion allele *unc-22(bch26)*, sgRNA #1 guided Cas9 to the plasmid, resulting in a cut, but there was no evidence of a cut in the target site of the sgRNA #1 in the genomic locus. sgRNA #2 guided Cas9 to cut the genomic locus, which was repaired by insertion of the linearized plasmid, as shown.

We explored the possibility of targeting insertion of a bright *Peft-3*::*gfp*::*h2b*::*tbb-2* 3′UTR construct into a genomic locus through NHEJ (Figure 2). We cloned a previously tested sgRNA targeting site for *unc-22*, whose corresponding sgRNA cuts efficiently, and its immediate genomic context, into a plasmid containing *Peft-3*::*gfp*::*h2b*::*tbb-2* 3′UTR and a *C. briggsae unc-119* rescuing fragment, upstream of the transgenes. The 30 nucleotide length was chosen as the immediate context was shown to affect efficiency of sgRNA cutting the genomic site in mammalian cells (Doench *et al.* 2014). We reasoned that concomitant cutting of the plasmid and the genomic target might, in some cases, result in NHEJ-mediated knock-in of the plasmid into the genomic locus, as was shown by Auer *et al.* (2014) in zebrafish. We microinjected 30 *unc-119(ed3)* animals with a mixture containing Cas9, two sgRNAs targeting *unc-22*, the NHEJ targeting construct, and a mixture of red fluorescent markers (Frøkjær-Jensen *et al.* 2012) and screened them analogously to MosSCI (Frøkjaer-Jensen *et al.* 2008; Frøkjær-Jensen *et al.* 2012): integration of the transgene results in loss of red fluorescent markers, but non-Unc-119 animals are present on plates. One F_2_ animal expressed bright, ubiquitous green fluorescent protein (GFP) and no red markers, and twitched when moving, which is a phenotype of *unc-22(lof)* alleles (Moerman *et al.* 1988). The resulting strain was termed *unc-22(bch26)*. We backcrossed this mutant to the wild-type parent strain twice and performed genetic linkage analysis. 189/189 Unc-22 animals segregating from *unc-22(bch26)/+* parents give rise to only GFP positive progeny, showing that *Peft-3*::*gfp*::*h2b*::*tbb-2* 3′UTR insertion is closely linked to the *unc-22* locus. Subsequent PCR analysis and sequencing of the mutant revealed that the insertion disrupts the *unc-22* locus, as designed (Figure S1 and Figure S2).

On the basis of the brightness of green fluorescence in the *unc-22(bch26)* animals, we repeated the injection into 30 N2 animals, reasoning that screening by Unc-119 phenotype rescue was not essential. In this experiment, we also identified one GFP- but not mCherry-expressing, twitcher animal upon chunking starved P_0_ plates. However, this integration was not genetically linked to *unc-22*—perhaps inserting into an off-target site of one of the sgRNAs used—and was not analyzed further.

To show that this method could be used to target a different gene—one we have previously unsuccessfully tried to modify through homologous recombination—we cloned an sgRNA targeting site for *lin-41* and its immediate genomic context into the *Peft-3*::*gfp*::*h2b*::*tbb-2* 3′UTR plasmid, 5′ to the transgene. This plasmid did not contain *unc-119* rescuing sequences. As described previously, we injected the mix, this time containing just a single sgRNA, into gonads of 30 N2 animals and obtained one candidate GFP-expressing, but not mCherry-expressing, animal at the F_2_ stage. This animal segregated two kinds of GFP-positive progeny: sickly, dumpy animals that often died in early adulthood or were sterile (Figure S1B)—*lin-41* loss-of-function candidates (Slack *et al.* 2000)—and apparent wild-type animals. It also segregated nonfluorescent, wild-type animals, so it appeared to have been heterozygous for the insert. We termed it *lin-41(bch28)*, backcrossed it to the wild-type parent strain and then mated it with *lin-41(xe8)* mutants, which have a partial 3′ UTR deletion, resulting in inability of *lin-41* to be down-regulated by *let-7* (Ecsedi *et al.* 2015). Heterozygous *lin-41(xe8)/lin-41(bch28 [Peft-3*::*gfp*::*h2b*::*tbb-2 3′ UTR])* animals are fertile and segregate GFP and non−GFP-expressing animals. All 44 of the non-GFP-expressing animals burst after vulva eversion and are homozygous for the *xe8* deletion (M. Rausch, unpublished data). On sequencing analysis of the *lin-41* locus in the *lin-41(bch28)* animals, we observe a complex insert, containing sequences from at least two copies of the *Peft-3*::*gfp*::*h2b*::*tbb-2* 3′UTR plasmid, one of them truncated (Figure S1 and Figure S3). The insertion occurred in the targeted sgRNA binding site, as designed.

This approach potentially can be generalized to targeting any site in the *C. elegans* genome. It requires very simple cloning of a 30-bp sgRNA sequence by oligonucleotide hybridization and Gibson assembly into a linearized vector, followed by microinjection and screening, analogous to MosSCI. Frøkjær-Jensen *et al.* (2014) have shown that *Peft-3*::*tdTomato*::*h2b* single-copy inserts are visible under a dissecting microscope, so a similar approach to a balancer labeled with red fluorescence should also be feasible. As there are still regions in the *C. elegans* genome without a balancer, and conventional balancers can break (Edgley *et al.* 2006), this method might simplify and accelerate progress for researchers whose experiments depend on successful balancing. We note that Frøkjær-Jensen *et al.* (2014) have recently made available a large set of fluorescent insertions. Alone or in combination with genome editing, they can serve as balancers. However, our NHEJ-based method can be used when a very stable, fluorescently-labeled balancer is desired, as recombination between the balanced locus and the fluorescent marker should be virtually absent.

In conclusion, we show that oligonucleotides, double- and single-stranded circular DNA are comparable templates for homologous recombination-based changes of a few nucleotides upon Cas9 cleavage, and that oligonucleotide-templated repair appears to depend on polarity of the oligonucleotides. Changes in three genes occurred more efficiently when the template oligonucleotide was oriented in the sense direction to the gene transcription, through an unknown mechanism. We also recommend screening for recombination events in the F_2_ generation, because the most widely used constructs at this time, where the nuclease is expressed from the ubiquitous *eft-3* promoter, also lead to somatic recombination in the F_1_ generation. For all kinds of templates, we observe preferential recombinational repair in those progeny that will not inherit the DNA transgene array. Finally, we show that exogenous DNA can be inserted into precise loci of the genome by NHEJ through a simple method.

## Supplementary Material

Supporting Information
