# Supplementary material for: CRISPR/Cas9 Genome Editing in Caenorhabditis elegans: Evaluation of Templates for Homology-Mediated Repair and Knock-Ins by Homology-Independent DNA Repair
Source: G3 (Bethesda). 2015 Jun 3;5(8):1649–56. doi: 10.1534/g3.115.019273 (PMC4528321; doi:10.1534/g3.115.019273)
Supplement: Supporting Information [file supp_5_8_1649__index.html]

CRISPR/Cas9 Genome Editing in Caenorhabditis elegans: Evaluation of Templates for Homology-Mediated Repair and Knock-Ins by Homology-Independent DNA Repair — Supporting Information 

# CRISPR/Cas9 Genome Editing in *Caenorhabditis elegans*: Evaluation of Templates for Homology-Mediated Repair and Knock-Ins by Homology-Independent DNA Repair

## Supporting Information for Katic, Xu, and Ciosk, 2015

**Files in this Data Supplement:**

- Supporting Information - Tables S1-S4 and Figures S1-S3 (PDF, 3 MB)
- Table S1 - Summary of comparisons of repair by sense and antisense oligonucleotides from this study and Ward (2015). (PDF, 69 KB)
- Table S2 - sgRNAs with complementarity of >20 nt to their target site can guide Cas9. (PDF, 97 KB)
- Table S3 - Mutagenicity of four sgRNAs targeting *unc-22* correlate with predictions of the web tool based on the study of Doench *et al.* (2014). (PDF, 83 KB)
- Table S4 - sgRNAs with published efficiency in *C. elegans* and their scores according to the Doench *et al.* (2014) algorithm. (PDF, 131 KB)
- Figure S1 - Knock-in of a plasmid into a genomic locus upon non-homologous end joining-mediated repair of a Cas9/CRISPR lesion. (PDF, 2 MB)
- Figure S2 - Sequence of the 20th exon of the *unc-22* locus in a) N2 and b) in the *unc-22(bch26)* allele. (PDF, 82 KB)
- Figure S3 - Sequence of the *lin-41* locus in a) N2 (partial) and b) in the *lin-41(bch28)* allele (partial). (PDF, 88 KB)
