# Supplementary material for: CRISPR/Cas9 Genome Editing in Caenorhabditis elegans: Evaluation of Templates for Homology-Mediated Repair and Knock-Ins by Homology-Independent DNA Repair
Source: G3 (Bethesda). 2015 Jun 3;5(8):1649–56. doi: 10.1534/g3.115.019273 (PMC4528321; doi:10.1534/g3.115.019273)
Supplement: Supporting Information [file supp_g3.115.019273_019273SI.pdf]

G3·Genes | Genomes | Genetics

CRISPR/Cas9 genome editing in *Caenorhabditis elegans*: evaluation of templates for homology-mediated repair and knock-ins by homology-independent DNA repair

Iskra Katic, Lan Xu, and Rafal Ciosk

Friedrich Miescher Institute for Biomedical Research, CH-4058 Basel, Switzerland

Corresponding author:

Iskra Katic
Friedrich Miescher Institute for Biomedical Research
Maulbeerstrasse 66
CH-4058 Basel
Switzerland
Phone: +41 61 696 31 52
iskra.katic@fmi.ch

DOI: 10.1534/g3.115.019273

Table S1. Summary of comparisons of repair by sense and antisense oligonucleotides from this study and Ward (2015)

gene	sgRNA binds coding strand of locus	preferred oligo template	reference
pha-1	yes	sense	Ward (2015)
nhr-23	no	sense	Ward (2015)
sqt-1 (sgRNA #1)	yes	sense	this study
sqt-1 (sgRNA #2)	no	sense	this study
sqt-1 (sgRNA #3)	yes	sense	this study
lin-12	no	sense	this study
dpy-10	yes	sense	this study

Table S2. sgRNAs with complementarity of >20 nt to their target site can guide Cas9.

Efficiency of repair by an oligonucleotide in the sense direction in experiments using Cas9 and each sgRNA in turn, to yield *sqt-1(sc1)/+* roller animals. *Relative yield of recombinants in each experiment is calculated by dividing the number of mutant F_1s with heritable mutations from each experiment by the number of animals positive for the fluorescent transformation marker resulting from the experiment, as a measure of microinjection efficiency.

sgRNA	recombinants	fluorescent	relative yield of recombi nants	SD (n=3)
pIK148<br>5′ AUGUGGAGUUGGGGUAGCGU 3′	62	248	32%	26%
pIK172<br>5′ AUCAGCAUGUGGAGUUGGGGUAGCGU 3′	24	175	13%	5%
pIK173<br>5′ AUCCAUCAGCAUGUGGAGUUGGGGUAGCGU 3′	24	537	4%	5%

Table S3. Mutagenicity of four sgRNAs targeting *unc-22* correlate with predictions of the web tool based on the study of Doench *et al.* (2014)

10 N2 animals were microinjected with a mix containing Cas9, one of the sgRNAs and a GFP marker. GFP positive F_1 animals were isolated and scored for segregation of Unc-22 twitcher animals. pIK214 sgRNA was modified so that the beginning nucleotide was A (underlined) instead of U, for improved transcription.

sgRNA	Unc-22/fluorescent (%)	sgRNA design tool score
pIK206<br>5′ GACAAGCCGAAACCACCAAA 3′	16/99 (16)	0.82
pIK199<br>5′ GCUCCAUUGGUAUGGUACCG 3′	86/97 (89)	0.76
pIK214<br>5′ <u>A</u>UCCACGAUUCAUUAUUGAAA 3′	1/94 (1)	0.05
pIK207<br>5′ AAACAAAUUUCCAGUAUGCC 3′	14/226 (6)	0.04

Table S4. sgRNAs with published efficiency in *C. elegans* and their scores according to the *Doench et al.* (2014) algorithm

sgRNAs were analyzed as in Farboud and Meyer (2015); sgRNAs from that study and ours were added and analyzed by the Doench *et al*. (2014) algorithm, at http://www.broadinstitute.org/rnai/public/analysis-tools/sgrna-design

gene	sequence	sgRNA design tool score	efficiency (%)	reference
Y62E10A.17	ATACGCACCGATGCTCTCCG	0.89	14	Farboud and Meyer, 2015
unc-22	GACAAGCCGAAACCACCAAA	0.82	16	this study
avr-14	GATTGGAGAGTTAGACCACG	0.79	20	Kim *et al*., 2014
unc-22	GCTCCATTGGTATGGTACCG	0.76	89	this study
fox-1	ATTACAGTGAAGTACAGCGG	0.75	21	Farboud and Meyer, 2015
dpy-11	GCAAGGATCTTCAAAAAGCA	0.75	10	Waaijers *et al*., 2013
lon-2	GGGAAACTATACCCTCACTG	0.68	30	Kim *et al*., 2014
fox-1	ACAATTACAGTGAAGTACAG	0.67	0	Farboud and Meyer, 2015
rol-1	GGAGGTTGACTCCAATACTA	0.5	1.4	Waaijers *et al*., 2013
unc-4	GTTATCGTCATCCGGTGACG	0.46	10	Kim *et al*., 2014
pie-1	GCTGAGAGAAGAATCCATCG	0.44	15	Kim *et al*., 104
sex-1	AACATTTCCACAACGAGAGG	0.43	51	Farboud and Meyer, 2015
fox-1	AATATCGTTTACCAAAACGG	0.42	13	Farboud and Meyer, 2015
Y61A9LA.1	GGATGGATGTGTAGTCAATT	0.37	18	Friedland *et al*., 2013
sex-1	TGGAACATTTCCACAACGAG	0.35	8	Farboud and Meyer, 2015
pie-1	GTTGAGTGCAGCCATTTGCT	0.35	5	Kim *et al*., 2014
pie-1	GGACAAAGAGAGGGGGTGAG	0.34	7.5	Kim *et al*., 2014
sex-1	AACGGATGAGAATCTGACAA	0.28	21	Farboud and Meyer, 2015
xol-1	AGCGATTTCTGGCGATTGGG	0.28	10	Farboud and Meyer, 2015
dpy-11	GATGCTTGTAGTCTGGAACT	0.25	0	Kim *et al*., 2014
klp-12	GATCCACAAGTTACAATTGG	0.23	80.3	Friedland *et al*., 2013
pie-1	GGCTCAGATTGACGAGGCGC	0.21	24	Kim *et al*., 2014
sex-1	CGCACCGATGCTCTCCGAGG	0.2	54	Farboud and Meyer, 2015
fox-1	ATATGAGGGGAGTGAGGCGG	0.18	29	Farboud and Meyer, 2015
unc-22	GAACCCGTTGCCGAATACAC	0.16	5	Kim *et al*., 2014
sex-1	AAACCTGCCTCCTCTCGTTG	0.16	0	Farboud and Meyer, 2015
ben-1	GGATATCACTTCCCAGAACT	0.13	0	Kim *et al*., 2014
lir-2	TTGACTCGTTCCATTTCAGC	0.11	0	Farboud and Meyer, 2015
C35E7.6	GGGCACCATACCGAGTGATG	0.1	100	Kim *et al*., 2014
fox-1	TTGAATATCGTTTACCAAAA	0.1	0	Farboud and Meyer, 2015
lin-5	GGAGCTTACTGAGACTCTTC	0.09	20.8	Waaijers *et al*., 2013
bli-2	GGATTTGCTGCTACTGAATC	0.09	0	Kim *et al*., 2014
avr-14	GAATATTGAAAGACTATGAT	0.08	10	Kim *et al*., 2014
dpy-13	GGACATTGACACTAAAATCA	0.08	0.5	Friedland *et al*., 2013
cpsf-2	CACTTTCAATTTGATAATGG	0.06	54	Farboud and Meyer, 2015
fox-1	CATTTGATATGAGGGGAGTG	0.06	20	Farboud and Meyer, 2015
unc-119	GTTATAGCCTGTTCGGTTAC	0.05	4.9	Waaijers *et al*., 2013
unc-22	(A)TCCACGATTCATTATTGAAA	0.05	1	this study

bli-2	GATGGACGGGATGGTAGAGA	0.05	0	Kim *et al*., 2014
lir-2	AATCAGCCGAGATGTAAGTT	0.05	0	Farboud and Meyer, 2015
Y62E10A.17	CGCACCGATGCTCTCCGAGG	0.04	57	Farboud and Meyer, 2015
unc-22	AAACAAATTTCCAGTATGCC	0.04	6	this study
cpsf-2	AAACACTTTCAATTTGATAA	0.03	0	Farboud and Meyer, 2015
xol-1	TCTAGCGATTTCTGGCGATT	0.01	0	Farboud and Meyer, 2015
cpsf-2	GTGGTTGGGATGAGCGATTC	0.01	0	Farboud and Meyer, 2015
lir-2	GGCTGATTTTCGCAGTTCGG	no score	72	Farboud and Meyer, 2015
vet-2	GTTGGATCATAGGATACCGG	no score	38	Kim et al., 2014
unc-119	GAATTTTCTGAAATTAAAGA	no score	3.7	Friedland et al., 2013
lir-2	CTCGGCTGATTTTCGCAGTT	no score	1	Farboud and Meyer, 2015
unc-22	GCCTTTGCTTCGATTTTCTT	no score	0	Kim et al., 2014
ben-1	GGGAGAAAGTGATTTGCAGT	no score	0	Kim et al., 2014
unc-32	GATAGGAAGCATCAGATTGA	no score	0	Kim et al., 2014
unc-32	GTTGCTGAACTGGGAGAGCT	no score	0	Kim et al., 2014

Supplementary Figure 1

A

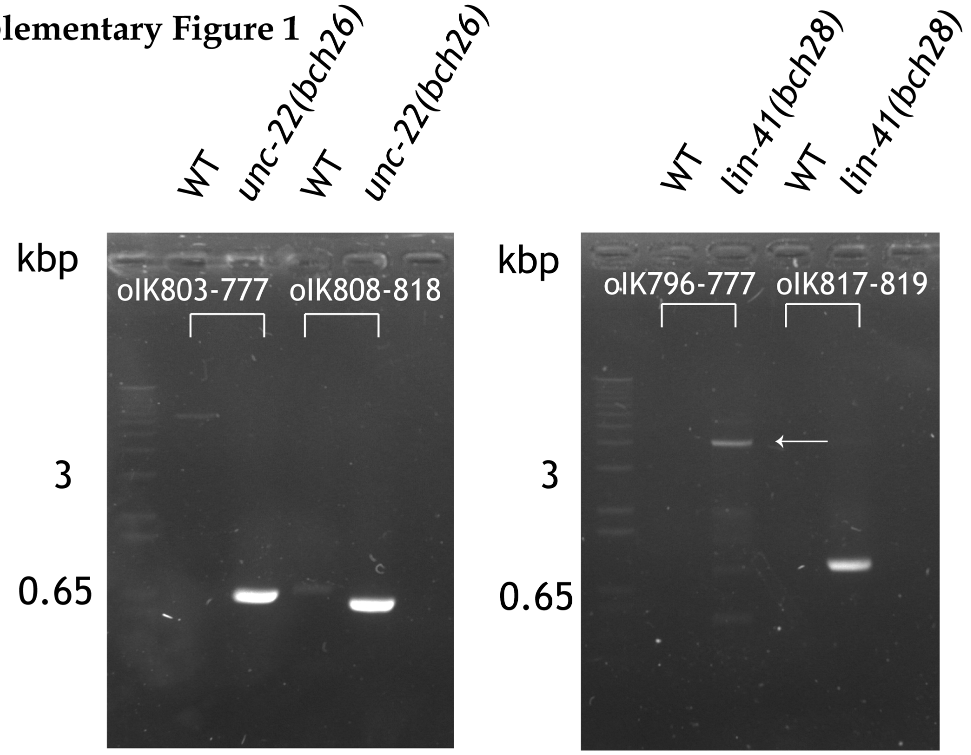

B

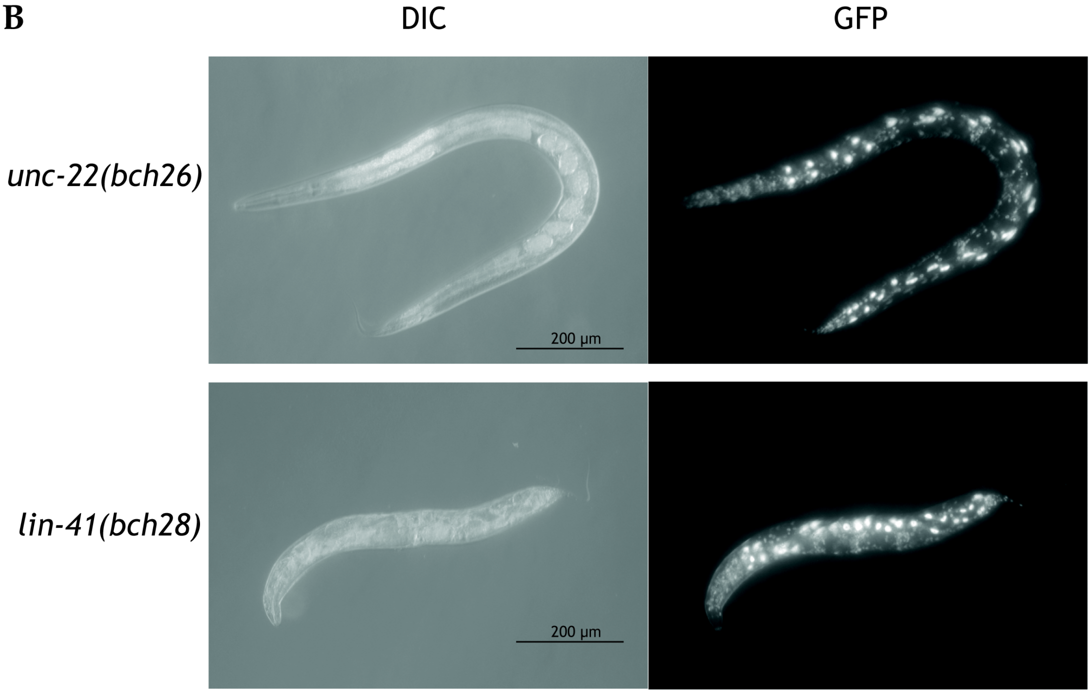

Figure S1. **Knock-in of a plasmid into a genomic locus upon non-homologous end joining-mediated repair of a Cas9/CRISPR lesion.**

A) PCR products spanning the junctions of *Peft-3::gfp::h2b::tbb-2 3'UTR* –containing plasmids into *unc-22* and *lin-41* loci. PCR primers are described in Materials and Methods. The arrow shows the oIK796-777 PCR product from *lin-41(bch28)* genomic lysates that was sequenced.
B) GFP expression from the *Peft-3::gfp::h2b::tbb-2 3'UTR*-containing plasmids in the *unc-22(bch26)* and *lin-41(bch28)* mutants.

Figure S2.

a) **Sequence of the 20th exon of the *unc-22* locus in N2**

genomic sgRNA site (**PAM** bold)

AACCGCCGATGAAGTACAGTTTCCTGAACCCGTTGCCGAATACACAGGAGATCTACCGTACT
AAGCAAGCAGTGCTCACATGTAAAGTGAACACACCACGTGCTCCATTGGTATGGTACCG**TGG**
AAGCAAGGCTATTCAAGAAGGAGATCCACGATTCATTATTGAAAAGGATGCCGTCGGTCGTT
GTACACTTACAATCAAGGAAGTTGAGGAAGACGATCAAGCTGAATGGACTGCTAGAATCACA
CAAGACGTGTTCTCAAAGGTTCAAGTGTACGTTGAGGAGCCACGGCATACATTCGTTGTTCC
AATGAAGTCGCAAAAAGTCAACGAAAGTGATTTGGCAACATTGGAGACTGATGTTAACGACA
AGGATGCTGAAGTTGTTTGGTGGCATGATGGAAAGAGAATCGATATTGATGGAGTGAAATTC
AAGGTTGAATCTTCAAACAGAAAGAGAAGACTTATTATCAATGGAGCTAGAATTGAAGATCA
TGGAGAGTATAAGTGTACAACTAAGGATGATAGAACTATGGCTCAGCTCATCGTTGATGCTA
AGAATAAGTTCATCGTTGCTCTCAAAGACACTGAAGTTATTGAGAAGGATGATGTTACATTG
ATGTGTCAGACAAAGGACACAAAAACTCCTGGAATTTGGTTCCGTAATGGAAAACAAATTTC
CAGTATGCCCGGAGGAAAGTTCGAAACTCAATCGAGAAACGGAACTCATACTCTTAAAATCG
GAAAGATCGAGATGAACGAGGCTGATGTTTATGAAATCGATCAGGCAGGACTACGTGGATCT
TGCAATGTGACTGTTCTCGAGGCAGAAAAGCGTCCAATTCTCAACTGGAAGCCAAAGAAAAT
CGAAGCAAAGGCTGGAGAACCATGTGTTGTGAAGGTTCCATTCCAAATCAAGGGAACACGAC
GTGGAGATCCAAAGGCTCAAATTCTGAAGAATGGAAAGCCAATCGATGAAGAAATGAGAAAG
CTAGTTGAAGTTATTATCAAGGATGATGTGGCTGAGATTGTTTTCAAAAATCCACAACTTGC
TGATACAGGAAAGTGGGCTCTCGAACTCGGAAACTCGGCTGGAACAGCACTTGCTCCATTCG
AGTTGTTCGTTAAGGACAAGCCGAAACCACCAAAGGGTCCACTTGAAACCAAGAATGTTACT
GCTGAAGGTCTTGATCTCGTCTGGGGAACTCCAGATCCAGATGAGGGAGCTCCAGTTAAAGC
ATACATCATTGAAATGCAAGAGGGAAGAAGTGGAAACTGGGCTAAAGTTGGAGAGACTAAGG
GAACAGACTTCAAGGTTAAGGATCTTAAAGAACATGGAGAATACAAGTTCAGAGTCAAGGCT
CTTAATGAATGCGGACTCTCTGATCCACTCACAGGAGAATCTGTTCTTGCCAAAAATCCATA
CGGCGTTCCTGGAAAACCAAAGAACATGGACGCAATTGATGTTGACAAGGATCACTGTACCC
TTGCATGGGAACCGCCAGAGGAGGATGGAGGTGCTCCAATCACTGGTTACATCATTGAAAGA
AGAGAGAAGTCCGAGAAAGATTGGCATCAAGTTGGACAGACCAAACCAGATTGTTGTGAACT
GACTGATAAGAAGGTTGTCGAAGATAAGGAATACTTGTACAGAGTAAAAGCAGTCAACAAGG
CTGGACCAGGAGACCCATGTGATCATGGAAAGCCAATCAAGATGAAAGCCAAGAAAGCTTCT
CCAGAATTCACTGGTGGAGGCATCAAGGATCTTCGTCTTAAGGTCGGAGAAACTATCAAGTA
CGACGTTCCAATTTCTGGAGAACCACTCCCAGAATGTCTTTGGGTGGTTAATGGAAAACCAC
TGAAGGCTGTTGGAAGAGTCAAGATGTCTTCTGAAAGAGGAAAGCATATCATGAAGATCGAA
AATGCAGTTCGTGCTGATTCCGGAAAGTTCACTATCACTTTGAAGAACTCTTCTGGCTCATG
CGACTCGACCGCCACGGTCACTGTCGTTGGAAGACCAACTCCACCAAAGGGTCCACTCGATA
TTGCTGATGTTTGTGCCGATGGTGCAACCCTTTCCTGGAATCCTCCAGATGATGATGGAGGT
GATCCACTCACAGGATACATCGTTGAAGCTCAAGATATGGACAACAAGGGAAAATACATTGA
AGTTGGAAAGGTTGATCCAAACACCACTACCCTCAAAGTTAATGGACTCCGTAACAAGGGAA
ATTACAAGTTCCGCGTGAAGGCAGTCAACAACGAAGGAGAATCTGAGCCACTTTCTGCTGAT
CAGTACACTCAGATCAAGGATCCTTGGGATGAACCAGGAAAGCCTGGAAGACCAGAAATTAC
CGATTTCGATGCGGATAGAATTGACATTGCCTGGGAGCCACCACACAAAGATGGAGGAGCTC
CAATCGAGGAGTATATTGTCGAAGTTCGTGATCCAGATACCAAAGAATGGAAGGAAGTCAAG
AGAGTTCCAGACACCAATGCATCAATTTCTGGATTGAAGGAAGGAAAGGAATATCAGTTCAG
AGTTCGGGCTGTTAACAAGGCTGGGCCTGGACAACCTTCCGAACCATCAGAGAAGCAATTGG
CTAAGCCAAAATTCA

b) Sequence of the 20th exon of the *unc-22* locus in the *unc-22(bch26)* allele

remainder of the genomic sgRNA site
unknown
Peft-3 in forward orientation
remainder of the sgRNA site in the *Peft-3::gfp::h2b::tbb-2* 3'UTR plasmid
plasmid sequence

AACCGCCGATGAAGTACAGTTTCCTGAACCCGTTGCCGAATACACAGGAGATCTACCGTACT
AAGCAAGCAGTGCTCACATGTAAAGTGAACACACCACGTGCTCCATTGGTATGGGTCCTTTG
TATAGAAAAGGGTCCTTTGTATAGAAAAGTTGGCACCTTTGGTCTTTTATTGTCAACTTCCA
TTGGTTCTTCCATTGTTTCTGTTAAATTAATGAATTTTTCATAAAATAAAGACATTATACAA
TATAAAAATGAAGAATTTATTGAAAATAAACTG[UNKNOWN SEQUENCE]
CAGATGCGTAAGGAGAAAATACCGCATCAGGCGCCATTCGCCATTCAGGCTGCGCAACTGTT
GGGAAGGGCGATCGGTGCGGGCCTCTTCGCTATTACGCCAGCTGGCGAAAGGGGGATGTGCT
GCAAGGCGATTAAGTTGGGTAACGCCAGGGTTTTCCCAGTCACGACGTTGTAAAACGACGGC
CAGTGAATTCGAGCTCGGTACCTCGCGAATGCATCTATAAG
GACAAGCCGAAACCACCGGAAGCAAGGCTATTCAAGAAGGAGATCCACGATTCATTATTGAA
AAGGATGCCGTCGGTCGTTGTACACTTACAATCAAGGAAGTTGAGGAAGACGATCAAGCTGA
ATGGACTGCTAGAATCACACAAGACGTGTTCTCAAAGGTTCAAGTGTACGTTGAGGAGCCAC
GGCATACATTCGTTGTTCCAATGAAGTCGCAAAAAGTCAACGAAAGTGATTTGGCAACATTG
GAGACTGATGTTAACGACAAGGATGCTGAAGTTGTTTGGTGGCATGATGGAAAGAGAATCGA
TATTGATGGAGTGAAATTCAAGGTTGAATCTTCAAACAGAAAGAGAAGACTTATTATCAATG
GAGCTAGAATTGAAGATCATGGAGAGTATAAGTGTACAACTAAGGATGATAGAACTATGGCT
CAGCTCATCGTTGATGCTAAGAATAAGTTCATCGTTGCTCTCAAAGACACTGAAGTTATTGA
GAAGGATGATGTTACATTGATGTGTCAGACAAAGGACACAAAAACTCCTGGAATTTGGTTCC
GTAATGGAAAACAAATTTCCAGTATGCCCGGAGGAAAGTTCGAAACTCAATCGAGAAACGGA
ACTCATACTCTTAAAATCGGAAAGATCGAGATGAACGAGGCTGATGTTTATGAAATCGATCA
GGCAGGACTACGTGGATCTTGCAATGTGACTGTTCTCGAGGCAGAAAAGCGTCCAATTCTCA
ACTGGAAGCCAAAGAAAATCGAAGCAAAGGCTGGAGAACCATGTGTTGTGAAGGTTCCATTC
CAAATCAAGGGAACACGACGTGGAGATCCAAAGGCTCAAATTCTGAAGAATGGAAAGCCAAT
CGATGAAGAAATGAGAAAGCTAGTTGAAGTTATTATCAAGGATGATGTGGCTGAGATTGTTT
TCAAAAATCCACAACTTGCTGATACAGGAAAGTGGGCTCTCGAACTCGGAAACTCGGCTGGA
ACAGCACTTGCTCCATTCGAGTTGTTCGTTAAGGACAAGCCGAAACCACCAAAGGGTCCACT
TGAAACCAAGAATGTTACTGCTGAAGGTCTTGATCTCGTCTGGGGAACTCCAGATCCAGATG
AGGGAGCTCCAGTTAAAGCATACATCATTGAAATGCAAGAGGGAAGAAGTGGAAACTGGGCT
AAAGTTGGAGAGACTAAGGGAACAGACTTCAAGGTTAAGGATCTTAAAGAACATGGAGAATA
CAAGTTCAGAGTCAAGGCTCTTAATGAATGCGGACTCTCTGATCCACTCACAGGAGAATCTG
TTCTTGCCAAAAATCCATACGGCGTTCCTGGAAAACCAAAGAACATGGACGCAATTGATGTT
GACAAGGATCACTGTACCCTTGCATGGGAACCGCCAGAGGAGGATGGAGGTGCTCCAATCAC
TGGTTACATCATTGAAAGAAGAGAGAAGTCCGAGAAAGATTGGCATCAAGTTGGACAGACCA
AACCAGATTGTTGTGAACTGACTGATAAGAAGGTTGTCGAAGATAAGGAATACTTGTACAGA
GTAAAAGCAGTCAACAAGGCTGGACCAGGAGACCCATGTGATCATGGAAAGCCAATCAAGAT
GAAAGCCAAGAAAGCTTCTCCAGAATTCACTGGTGGAGGCATCAAGGATCTTCGTCTTAAGG
TCGGAGAAACTATCAAGTACGACGTTCCAATTTCTGGAGAACCACTCCCAGAATGTCTTTGG
GTGGTTAATGGAAAACCACTGAAGGCTGTTGGAAGAGTCAAGATGTCTTCTGAAAGAGGAAA
GCATATCATGAAGATCGAAAATGCAGTTCGTGCTGATTCCGGAAAGTTCACTATCACTTTGA
AGAACTCTTCTGGCTCATGCGACTCGACCGCCACGGTCACTGTCGTTGGAAGACCAACTCCA
CCAAAGGGTCCACTCGATATTGCTGATGTTTGTGCCGATGGTGCAACCCTTTCCTGGAATCC
TCCAGATGATGATGGAGGTGATCCACTCACAGGATACATCGTTGAAGCTCAAGATATGGACA

ACAAGGGAAAATACATTGAAGTTGGAAAGGTTGATCCAAACACCACTACCCTCAAAGTTAAT
GGACTCCGTAACAAGGGAAATTACAAGTTCCGCGTGAAGGCAGTCAACAACGAAGGAGAATC
TGAGCCACTTTCTGCTGATCAGTACACTCAGATCAAGGATCCTTGGGATGAACCAGGAAAGC
CTGGAAGACCAGAAATTACCGATTTCGATGCGGATAGAATTGACATTGCCTGGGAGCCACCA
CACAAAGATGGAGGAGCTCCAATCGAGGAGTATATTGTCGAAGTTCGTGATCCAGATACCAA
AGAATGGAAGGAAGTCAAGAGAGTTCCAGACACCAATGCATCAATTTCTGGATTGAAGGAAG
GAAAGGAATATCAGTTCAGAGTTCGGGCTGTTAACAAGGCTGGGCCTGGACAACCTTCCGAA
CCATCAGAGAAGCAATTGGCTAAGCCAAAATTCA

Figure S3.

a) Sequence of the *lin-41* locus in N2 (partial)

lin-41 coding sequence
genomic sgRNA site (**PAM** bold)

gccacggttggcgaacgggtaaaaaggaagagccgatcgcctcgttactcaaggaaaaggct
cgacgtcgctggaggagggaaaagttggtttttaggataaaaaccaacttaaagtacaaaa
agagccggaaacggcgggaatgcgacgttggaaacgatcgtgatcttgaataaaggtgagga
acttttaaataacaaagcaaaatgcgaaaatatattaaaaattaaaaacgtcgaaaaaaaat
taataaaatttttttattcgaaaaatttgacctaccacaaatttttgactacttaatttt
ttgtttttgtttttattttcgaatattaaattgaaagagcggcatttttctgattttccca
tcaaaaatccttgtcatcagcagccctcgggccatttttctctcatttgttccattatttt
catataaaaagtgcactttttttgtgcttcactgactttttttgttcgattaattgttccatt
cgttctgaaaagtcaaaaaattcataagtattctaattgtagagtcatcgtttgccctttcc
actgataattaatcaacctttcagacttggaaaaagtgaaATGGCGACCATCGTGCCATGC
TCATTGGAGAAAGAAGAAGGAGCACCATCAGGACCTCGTCGGCTTCAAACTGAGATCGACG**T**
GGACGCCAACGACAGCGGAAACGAGCTGTCGATGGGCGGAAGCAGCAGTGAAGgtaacactt
gaaaattgaaaaaaaagcttccaacgaaacaacttcagGTGACTCAATGTCCCACCATCGCG
GTGAGCATTCACCAAACCATCATCATCAGGATAATCATCTTGGCTCGGGACCACCACCACCA
CAGTTCACTGGCTCACTATTTGACACTCCACCGTCAATGATTCAGTCACCACAGCAGCAACC
ACAGTTCCAGTTCAACACTGGATTCGGACTCGGACTCCCGCAAGACTCCTTTCGGTGCTCAG
TCTGCTCCAAGAGCTCGACGATTGGAGTGCTGCCGTTCGTTTGTGCGCACAAgtggggtttc
aagacaaattatcaaaatgacaattatttttagAACGTGCCAATCATGCTACCAGATGACAC
CGTCCTCGTACGATCGACGGGgtaaataaatatctcaactttctgtatgatttatgaatttt
agCTTGCAAGCTGTGTGGTGCCGTATCGACAGCCACCGCCAACTTTACATCGCAGATGTACC
TGTCTCCGACGCTTCCATCACCGCCACGTGGAGCTCTGATGTCGGATTGTTCGACACCAACG
ATGAATAATCACATTAATTgtatgttttaattctcaatttacaccgaagaataacccaaatt
ccagCATCAACTCCACTTCATCAACCTCGTGCATTCTCATTCTCACTCAGTGGAATGCCTGG
ATCACCGTCTCCAGTGATGGGTGCACGGATGCCATCGTCTGCTGGTGGGCTCATGATGCGTC
CAATTGGATTCCCCGACTCGGACAGCAGTTTGACATCATGGTCTCCATTGCAACAACCATCA
CAGCTTTCTATCAATAATCTGTCGAGCATCGGAGGACACCAGCAACAATCGCCGATGTTGAT
GCAAAATGTGTTTGATTCGTTGGCAGTGgtcagtttttatcaaatttcaaacagagaaaagt
acaattttgtggagtgtggagctataaacaaattcaatttcccgaatttatgatgttatata
ttatgtggtcgcaaatcgaaaaatggttcaatttacggtttcgccgtcaatacctaacgaga
cccaacttctggggtggacctcgattaaaaaatgagcatttttctacggccccataatatt
tcaaaaatcatttttttcgcaaaaaaaagttagcatttctctcttcccaagaaacatatttt
tctccctggcttcaataagttcaataagtctttttctaattttttttgctaaatgcgccct
cctgcgagctgaaattgcacctaattcctcgtattctttacagAACGATGATACTCCTGTAT
TCTCTCCACTCTCTCCAACGAATACTTCAATGCATATGCCACCATCATTGATGGCATCACCA
GATGTTCCAAAACATTCTGCAACCATAGCTCCACCACGTAATTCCATGTGCTCGACGCCACG
TCTTCAATTGGCAACACCAATGTCTTCACAATCACAGCAAACTTTTCCAATTCCTTCACCAC
TTGGATCTCAGCCACAACAACAACAGCCAATGGGTCCGATTCAATGTCAAGGATGCGAATCG
AAAATgttagttttccccctaaaacttcaaagataattaaattatatttaaatttttcagATC
GTTCGCCTACTGTATGCAATGTCAGGAAGCTTTGTGTATTCATTGTGTTCAAGCTCATCAGC
GTGTTCGAGCCACCAAACAACATGCGTTCGTTGAGCTTCAGCAGTTGATGGCTACTTTGgtt
agtttttatcttatcgtgtgttactacacgctttttgcgtctcaacacgacaattatttttt
tggaaattcataaaaagtatgcgcctttaaaacgtactgtaattaaacttttccattgtttt
tcattaaatatttgtatatttatttatcgaaaacctaaaattaaaaattaaaaccataaaaa
atcgatgaaaattcacagctacaaaaatttgaaactacagtactctttaaaggcgcacacct
ttttgtagtttacaaaaattgacattttgagacatgataccgtagtttgggagcaaaaaata

atatatgtggtctatattaatatcaccttgatccgtttcaagattctccacgtgcttgatga
cgtgaatcgtttgtaatcaggaatcataaaaagagtacgcaataatcataaatcaatgactc
ctgtcaatcaagaaaaaacacagaatcagtactttttggtgtatttttccgtttttaaatg
tcttatcagtaactgattgcaggattagaacacaaaaattggctgccgaccgttccactaca
ttagtaaaacttcttgaaaataacttttgaaaactatcaaggaaagtatttcagctcactt
ttagattttaaatgaaacttgtgtcagcagtttcacatttttcaaacaaaaccaagttcca
aaaagctcccagagtgtttgcttacctccaaacaccataaacatcaccaaaagcttgcaaaa
atccacatttcgaagaaaacaatttactgaaagcagtttcagagctgaatactttccttac
aagtttatagattttctatccgaatatgtccacgtactaggaaattcccatttttcaacat
ccaggcgttcgtttccctttttcttctccttcaaccacctttttcgccattattcacaagc
cagtcggagggccaatttagtgccgagagagagagaggtgtctgctcctcatccggtgctct
ttttgcactatagtattatttttgctcggcgaacacgtaattcctctaaaatctaaaagta
ttattttcagATGAGCCGTGCAGTTCAACCACAACAAGCACAGCAATACACTCAAAATGTTG
GAGGATCAGTTCGTCAAGCACTTGGATCAGTTGGAAGTGGAGATGgtatat

b)Sequence of the *lin-41* locus in the *lin-41(bch28)* allele (partial)

lin-41(bch28)

lin-41 coding sequence
remainder of the genomic sgRNA site
unknown
truncated GFP
tbb-2 3'UTR
Peft-3 in reverse orientation
sgRNA site (reverse and complement) in the *Peft-3::gfp::h2b::tbb-2* 3'UTR plasmid; one base is deleted between the nucleotides in red
plasmid sequence

gccacggttggcgaacgggtaaaaaggaagagccgatcgcctcgttactcaaggaaaaggct
cgacgtcgctggaggagggaaaagttggtttttaggataaaaaccaacttaaagtacaaaa
agagccggaaacggcgggaatgcgacgttggaaacgatcgtgatcttgaataaaggtgagga
acttttaaataacaaagcaaaatgcgaaaatatattaaaaattaaaaacgtcgaaaaaaaat
taataaaatttttttattcgaaaaatttttgacctaccacaaatttttttgactacttaatttt
ttgtttttgttttattttcgaatattaaattgaaagagcggcatttttctgattttccca
tcaaaaatccttgtcatcagcagccctcgggccatttttctctcatttttgttccattatttt
catataaaaagtgcactttttttgtgcttcactgactttttttgttcgattaattgttccatt
cgttctgaaaagtcaaaaaattcataagtattctaattgtagagtcatcgtttgccctttcc
actgataattaatcaacctttttcagacttggaaaaagtgaaATGGCGACCATCGTGCCATGC
TCATTGGAGAAAGAAGAAGGAGCACCATCAGGACCTCGTCGGgtgctcatcattggaacaag
accgtcgttgccaagccaaaggacggaaagaagagacgtcatgcccgcaaggaatcgtactc
cgtctacatctaccgtgttctcaagcaagttcacccagacaccggagtctcctccaaggcca
tgtctatcatgaactccttcgtcaacgatgtattcgaacgcatcgcttcggaagcttcccgt
cttgctcattacaacaaacgctcaacgatctcatcccgcgaaattcaaaccgctgtccgttt
gattctcccaggagaacttgccaagcacgccgtgtctgagggaaccaaggccgtcaccaagt
acacttccagcaagtaaacccagctttcttgtacaaagtgggataaatgcaaaatcctttca
agcattcccttcttctctatcactcttctttctttttgtcaaaaaattctctcgctaattta
tttgctttttttaatgttattattttatgacttttatagtcactgaaaagtttgcatctgag
tgaagtgaatgctatcaaaatgtgattctgtctgatgtactttcacaatctctcttcaattc

cattttgaagtgctttaaacccgaaaggttgagaaaaatgcgagcgctcaaatatttgtatt
gtgttcgttgagtgacccaacaaaaagaggaaactttattgtgccgccaagaaaaaagtctc
acaactatcggatcc[UNKNOWN
SEQUENCE]CTCTCGGCGGGAGTGTTGCATACTTTTTCTCTCTGGCAGTTTATTTTCAATAA
ATTCTTCATTTTTATATTGTATAATGTCTTTATTTTATGAAAAATTCATTAATTTAACAGAA
ACAATGGAAGAACCAATGGAAGTTGACAATAAAAGACCAAAGGTGCcaactttctatacaa
agatctagatgcattcgcgaggtaccgagctcg**CGTCCACGTGATCTCAGTTTGAAGCCGAC**
cactggccgtcgttttacaacgtcgtgactgggaaaaccctggcgttacccaacttaatcgc
cttgcagcacatccccctttcgccagctggcgtaatagcgaagaggcccgcaccgatcgccc
ttcccaacagttgcgcagcctgaatggcgaatggcgcctgatgcggtattttctccttacgc
atctgtgcggtatttcacaccgcatatggtgcactctcagtacaatctgctctgatgccgca
tagttaagccagccccgacacccgccaacacccgctgacgcgccctgacgggcttgtctgct
cccggcatccgcttacagacaagctgtgaccgtctccgggagctgcatgtgtcagaggtttt
caccgtcatcaccgaaacgcgcgagacgaaagggcctcgtgatacgcctatttttataggtt
aatgtcatgataataatggtttcttagacgtcaggtggcacttttcggggaaatgtgcgcgg
aacccctatttgtttatttttctaaatacattcaaatatgtatccgctcatgagacaataac
cctgataaatgcttcaataatattgaaaaaggaagagtatgagtattcaacatttccgtgtc
gcccttattcccttttttgcggcattttgccttcctgtttttgctcacccagaaacgctggt
gaaagtaaaagatgctgaagatcagttgggtgcacgagtgggttacatcgaactggatctca
acagcggtaagatccttgagagttttcgccccgaagaacgttttccaatgatgagcactttT
CGATGGGCGGAAGCAGCAGTGAAGgtaacacttgaaaattgaaaaaaaagcttccaacgaaa
caacttcagGTGACTCAATGTCCCACCATCGCGGTGAGCATTCACCAAACCATCATCATCAG
GATAATCATCTTGGCTCGGGACCACCACCACCACAGTTCACTGGCTCACTATTTGACACTCC
ACCGTCAATGATTCAGTCACCACAGCAGCAACCACAGTTCCAGTTCAACACTGGATTCGGAC
TCGGACTCCCGCAAGACTCCTTTCGGTGCTCAGTCTGCTCCAAGAGCTCGACGATTGGAGTG
CTGCCGTTCGTTTGTGCGCACAAgtggggtttcaagacaaattatcaaaatgacaattattt
ttagAACGTGCCAATCATGCTACCAGATGACACCGTCCTCGTACGATCGACGGGgtaaataa
atatctcaactttctgtatgatttatgaattttagCTTGCAAGCTGTGTGGTGCCGTATCGA
CAGCCACCGCCAACTTTACATCGCAGATGTACCTGTCTCCGACGCTTCCATCACCGCCACGT
GGAGCTCTGATGTCGGATTGTTCGACACCAACGATGAATAATCACATTAATTgtatgtttta
attctcaatttacaccgaagaataacccaaattccagCATCAACTCCACTTCATCAACCTCG
TGCATTCTCATTCTCACTCAGTGGAATGCCTGGATCACCGTCTCCAGTGATGGGTGCACGGA
TGCCATCGTCTGCTGGTGGGCTCATGATGCGTCCAATTGGATTCCCCGACTCGGACAGCAGT
TTGACATCATGGTCTCCATTGCAACAACCATCACAGCTTTCTATCAATAATCTGTCGAGCAT
CGGAGGACACCAGCAACAATCGCCGATGTTGATGCAAAATGTGTTTGATTCGTTGGCAGTGg
tcagtttttatcaaatttcaaacagagaaaagtacaattttgtggagtgtggagctataaac
aaattcaatttcccgaatttatgatgttatatattatgtggtcgcaaatcgaaaaatggttc
aatttacggtttcgccgtcaatacctaacgagacccaacttctgggggtggacctcgattaa
aaaatgagcatttttctacggccccataatatttcaaaaatcatttttttcgcaaaaaaaag
ttagcatttctctcttcccaagaaacatatttttctccctggcttcaataagttcaataagt
ctttttctaatttttttttgctaaatgcgccctcctgcgagctgaaattgcacctaattcct
cgtattctttacagAACGATGATACTCCTGTATTCTCTCCACTCTCTCCAACGAATACTTCA
ATGCATATGCCACCATCATTGATGGCATCACCAGATGTTCCAAAACATTCTGCAACCATAGC
TCCACCACGTAATTCCATGTGCTCGACGCCACGTCTTCAATTGGCAACACCAATGTCTTCAC
AATCACAGCAAACTTTTCCAATTCCTTCACCACTTGGATCTCAGCCACAACAACAACAGCCA
ATGGGTCCGATTCAATGTCAAGGATGCGAATCGAAAATgttagttttccccctaaaacttcaa
agataattaaattatatttaaatttttcagATCGTTCGCCTACTGTATGCAATGTCAGGAAG
CTTTGTGTATTCATTGTGTTCAAGCTCATCAGCGTGTTCGAGCCACCAAACAACATGCGTTC
GTTGAGCTTCAGCAGTTGATGGCTACTTTGgttagtttttatcttatcgtgtgttactacac
gcttttttgcgtctcaacacgacaattattttttttggaaattcataaaaagtatgcgccttta
aaacgtactgtaattaaacttttccattgtttttcattaaatatttgtatatttatttatcg
aaaacctaaaattaaaaattaaaaccataaaaaatcgatgaaaattcacagctacaaaaatt

tgaaactacagtactctttaaaggcgcacacctttttgtagtttacaaaaattgacattttg
agacatgataccgtagtttgggagcaaaaaataatatatgtggtctatattaatatcacctt
gatccgtttcaagattctccacgtgcttgatgacgtgaatcgtttgtaatcaggaatcataa
aaagagtacgcaataatcataaatcaatgactcctgtcaatcaagaaaaaacacagaatcag
tactttttggtgtatttttccgtttttaaatgtcttatcagtaactgattgcaggattaga
acacaaaaattggctgccgaccgttccactacattagtaaaacttcttgaaaataacttttt
gaaaactatcaaggaaagtatttcagctcacttttagatttttaaatgaaacttgtgtcagc
agtttcacatttttcaaacaaaaccaagttccaaaaagctcccagagtgtttgcttacctcc
aaacaccataaacatcaccaaaagcttgcaaaaatccacatttcgaagaaaacaatttactg
aaagcagtttcagagctgaaatactttccttacaagtttatagatttttctatccgaatatg
tccacgtactaggaaattcccatttttcaacatccaggcgttcgtttcccttttcttctcc
ttcaaccacctttttcgccattattcacaagccagtcggagggccaatttagtgccgagag
agagagaggtgtctgctcctcatccggtgctctttttgcactatagtattatttttgctcg
gcgaacacgtaattcctctaaaatctaaaagtattattttcagATGAGCCGTGCAGTTCAAC
CACAACAAGCACAGCAATACACTCAAAATGTTGGAGGATCAGTTCGTCAAGCACTTGGATCA
GTTGGAAGTGGAGATGgtatat
