# Supplementary material for: CRISPR/Cas9 Genome Editing in Caenorhabditis elegans: Evaluation of Templates for Homology-Mediated Repair and Knock-Ins by Homology-Independent DNA Repair
Source: G3 (Bethesda). 2015 Jun 3;5(8):1649–56. doi: 10.1534/g3.115.019273 (PMC4528321; doi:10.1534/g3.115.019273)
Supplement: Supporting Information [file supp_g3.115.019273_TableS1.pdf]

Table S1. Summary of comparisons of repair by sense and antisense oligonucleotides from this study and Ward (2015)

gene	sgRNA binds coding strand of locus	preferred oligo template	reference
pha-1	yes	sense	Ward (2015)
nhr-23	no	sense	Ward (2015)
sqt-1 (sgRNA #1)	yes	sense	this study
sqt-1 (sgRNA #2)	no	sense	this study
sqt-1 (sgRNA #3)	yes	sense	this study
lin-12	no	sense	this study
dpy-10	yes	sense	this study
