# Supplementary material for: CRISPR/Cas9 Genome Editing in Caenorhabditis elegans: Evaluation of Templates for Homology-Mediated Repair and Knock-Ins by Homology-Independent DNA Repair
Source: G3 (Bethesda). 2015 Jun 3;5(8):1649–56. doi: 10.1534/g3.115.019273 (PMC4528321; doi:10.1534/g3.115.019273)
Supplement: Supporting Information [file supp_g3.115.019273_TableS2.pdf]

Table S2. sgRNAs with complementarity of >20 nt to their target site can guide Cas9.

Efficiency of repair by an oligonucleotide in the sense direction in experiments using Cas9 and each sgRNA in turn, to yield *sqt-1(sc1)/+* roller animals. *Relative yield of recombinants in each experiment is calculated by dividing the number of mutant F_1s with heritable mutations from each experiment by the number of animals positive for the fluorescent transformation marker resulting from the experiment, as a measure of microinjection efficiency.

sgRNA	recombinants	fluorescent	relative yield of recombi nants	SD (n=3)
pIK148<br>5′ AUGUGGAGUUGGGGUAGCGU 3′	62	248	32%	26%
pIK172<br>5′ AUCAGCAUGUGGAGUUGGGGUAGCGU 3′	24	175	13%	5%
pIK173<br>5′ AUCCAUCAGCAUGUGGAGUUGGGGUAGCGU 3′	24	537	4%	5%
