# Supplementary material for: CRISPR/Cas9 Genome Editing in Caenorhabditis elegans: Evaluation of Templates for Homology-Mediated Repair and Knock-Ins by Homology-Independent DNA Repair
Source: G3 (Bethesda). 2015 Jun 3;5(8):1649–56. doi: 10.1534/g3.115.019273 (PMC4528321; doi:10.1534/g3.115.019273)
Supplement: Supporting Information [file supp_g3.115.019273_TableS3.pdf]

Table S3. Mutagenicity of four sgRNAs targeting *unc-22* correlate with predictions of the web tool based on the study of Doench *et al*. (2014)

10 N2 animals were microinjected with a mix containing Cas9, one of the sgRNAs and a GFP marker. GFP positive F_1 animals were isolated and scored for segregation of Unc-22 twitcher animals. pIK214 sgRNA was modified so that the beginning nucleotide was A (underlined) instead of U, for improved transcription.

sgRNA	Unc-22/fluorescent (%)	sgRNA design tool score
pIK206<br>5′ GACAAGCCGAAACCACCAAA 3′	16/99 (16)	0.82
pIK199<br>5′ GCUCCAUUGGUAUGGUACCG 3′	86/97 (89)	0.76
pIK214<br>5′ AUCCACGAUUCAUUAUUGAAA 3′	1/94 (1)	0.05
pIK207<br>5′ AAACAAAUUUCCAGUAUGCC 3′	14/226 (6)	0.04
