# Supplementary material for: CRISPR/Cas9 Genome Editing in Caenorhabditis elegans: Evaluation of Templates for Homology-Mediated Repair and Knock-Ins by Homology-Independent DNA Repair
Source: G3 (Bethesda). 2015 Jun 3;5(8):1649–56. doi: 10.1534/g3.115.019273 (PMC4528321; doi:10.1534/g3.115.019273)
Supplement: Supporting Information [file supp_g3.115.019273_TableS4.pdf]

Table S4. sgRNAs with published efficiency in *C. elegans* and their scores according to the *Doench et al.* (2014) algorithm

sgRNAs were analyzed as in Farboud and Meyer (2015); sgRNAs from that study and ours were added and analyzed by the Doench *et al*. (2014) algorithm, at http://www.broadinstitute.org/rnai/public/analysis-tools/sgrna-design

gene	sequence	sgRNA design tool score	efficiency (%)	reference
Y62E10A.17	ATACGCACCGATGCTCTCCG	0.89	14	Farboud and Meyer, 2015
unc-22	GACAAGCCGAAACCACCAAA	0.82	16	this study
avr-14	GATTGGAGAGTTAGACCACG	0.79	20	Kim *et al*., 2014
unc-22	GCTCCATTGGTATGGTACCG	0.76	89	this study
fox-1	ATTACAGTGAAGTACAGCGG	0.75	21	Farboud and Meyer, 2015
dpy-11	GCAAGGATCTTCAAAAAGCA	0.75	10	Waaijers *et al*., 2013
lon-2	GGGAAACTATACCCTCACTG	0.68	30	Kim *et al*., 2014
fox-1	ACAATTACAGTGAAGTACAG	0.67	0	Farboud and Meyer, 2015
rol-1	GGAGGTTGACTCCAATACTA	0.5	1.4	Waaijers *et al*., 2013
unc-4	GTTATCGTCATCCGGTGACG	0.46	10	Kim *et al*., 2014
pie-1	GCTGAGAGAAGAATCCATCG	0.44	15	Kim *et al*., 104
sex-1	AACATTTCCACAACGAGAGG	0.43	51	Farboud and Meyer, 2015
fox-1	AATATCGTTTACCAAAACGG	0.42	13	Farboud and Meyer, 2015
Y61A9LA.1	GGATGGATGTGTAGTCAATT	0.37	18	Friedland *et al*., 2013
sex-1	TGGAACATTTCCACAACGAG	0.35	8	Farboud and Meyer, 2015
pie-1	GTTGAGTGCAGCCATTTGCT	0.35	5	Kim *et al*., 2014
pie-1	GGACAAAGAGAGGGGGTGAG	0.34	7.5	Kim *et al*., 2014
sex-1	AACGGATGAGAATCTGACAA	0.28	21	Farboud and Meyer, 2015
xol-1	AGCGATTTCTGGCGATTGGG	0.28	10	Farboud and Meyer, 2015
dpy-11	GATGCTTGTAGTCTGGAACT	0.25	0	Kim *et al*., 2014
klp-12	GATCCACAAGTTACAATTGG	0.23	80.3	Friedland *et al*., 2013
pie-1	GGCTCAGATTGACGAGGCGC	0.21	24	Kim *et al*., 2014
sex-1	CGCACCGATGCTCTCCGAGG	0.2	54	Farboud and Meyer, 2015
fox-1	ATATGAGGGGAGTGAGGCGG	0.18	29	Farboud and Meyer, 2015
unc-22	GAACCCGTTGCCGAATACAC	0.16	5	Kim *et al*., 2014
sex-1	AAACCTGCCTCCTCTCGTTG	0.16	0	Farboud and Meyer, 2015
ben-1	GGATATCACTTCCCAGAACT	0.13	0	Kim *et al*., 2014
lir-2	TTGACTCGTTCCATTTCAGC	0.11	0	Farboud and Meyer, 2015
C35E7.6	GGGCACCATACCGAGTGATG	0.1	100	Kim *et al*., 2014
fox-1	TTGAATATCGTTTACCAAAA	0.1	0	Farboud and Meyer, 2015
lin-5	GGAGCTTACTGAGACTCTTC	0.09	20.8	Waaijers *et al*., 2013
bli-2	GGATTTGCTGCTACTGAATC	0.09	0	Kim *et al*., 2014
avr-14	GAATATTGAAAGACTATGAT	0.08	10	Kim *et al*., 2014
dpy-13	GGACATTGACACTAAAATCA	0.08	0.5	Friedland *et al*., 2013
cpsf-2	CACTTTCAATTTGATAATGG	0.06	54	Farboud and Meyer, 2015
fox-1	CATTTGATATGAGGGGAGTG	0.06	20	Farboud and Meyer, 2015
unc-119	GTTATAGCCTGTTCGGTTAC	0.05	4.9	Waaijers *et al*., 2013
unc-22	(A)TCCACGATTCATTATTGAAA	0.05	1	this study

bli-2	GATGGACGGGATGGTAGAGA	0.05	0	Kim *et al*., 2014
lir-2	AATCAGCCGAGATGTAAGTT	0.05	0	Farboud and Meyer, 2015
Y62E10A.17	CGCACCGATGCTCTCCGAGG	0.04	57	Farboud and Meyer, 2015
unc-22	AAACAAATTTCCAGTATGCC	0.04	6	this study
cpsf-2	AAACACTTTCAATTTGATAA	0.03	0	Farboud and Meyer, 2015
xol-1	TCTAGCGATTTCTGGCGATT	0.01	0	Farboud and Meyer, 2015
cpsf-2	GTGGTTGGGATGAGCGATTC	0.01	0	Farboud and Meyer, 2015
lir-2	GGCTGATTTTCGCAGTTCGG	no score	72	Farboud and Meyer, 2015
vet-2	GTTGGATCATAGGATACCGG	no score	38	Kim et al., 2014
unc-119	GAATTTTCTGAAATTAAAGA	no score	3.7	Friedland et al., 2013
lir-2	CTCGGCTGATTTTCGCAGTT	no score	1	Farboud and Meyer, 2015
unc-22	GCCTTTGCTTCGATTTTCTT	no score	0	Kim et al., 2014
ben-1	GGGAGAAAGTGATTTGCAGT	no score	0	Kim et al., 2014
unc-32	GATAGGAAGCATCAGATTGA	no score	0	Kim et al., 2014
unc-32	GTTGCTGAACTGGGAGAGCT	no score	0	Kim et al., 2014
