# Supplementary material for: CRISPR/Cas9 Genome Editing in Caenorhabditis elegans: Evaluation of Templates for Homology-Mediated Repair and Knock-Ins by Homology-Independent DNA Repair
Source: G3 (Bethesda). 2015 Jun 3;5(8):1649–56. doi: 10.1534/g3.115.019273 (PMC4528321; doi:10.1534/g3.115.019273)
Supplement: Supporting Information [file supp_g3.115.019273_FigureS1.pdf]

Supplementary Figure 1

A

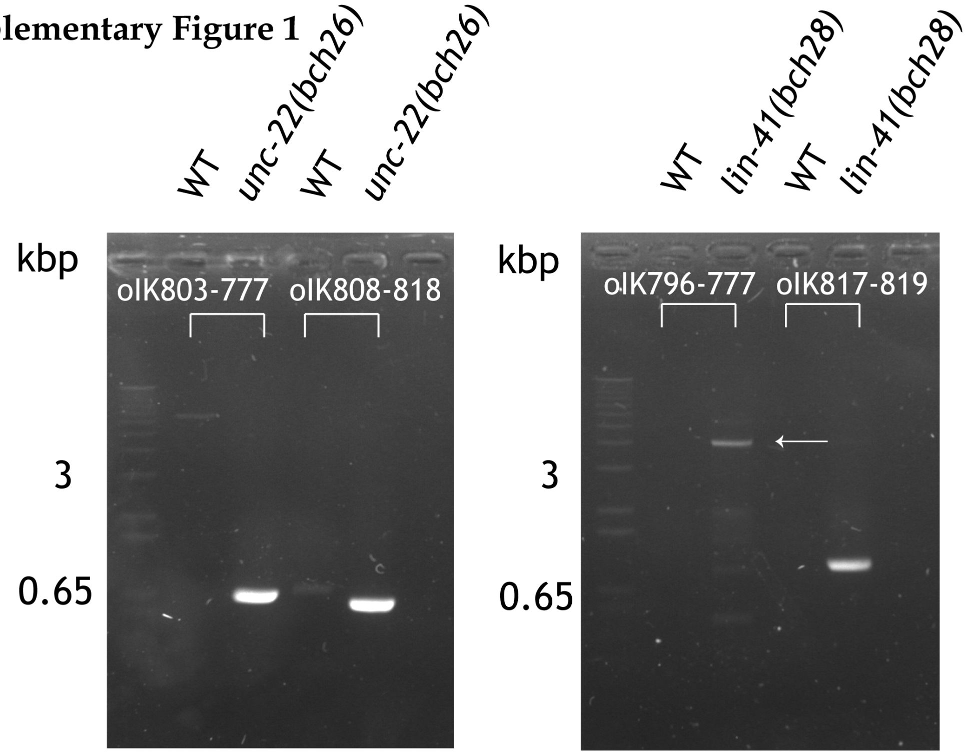

B

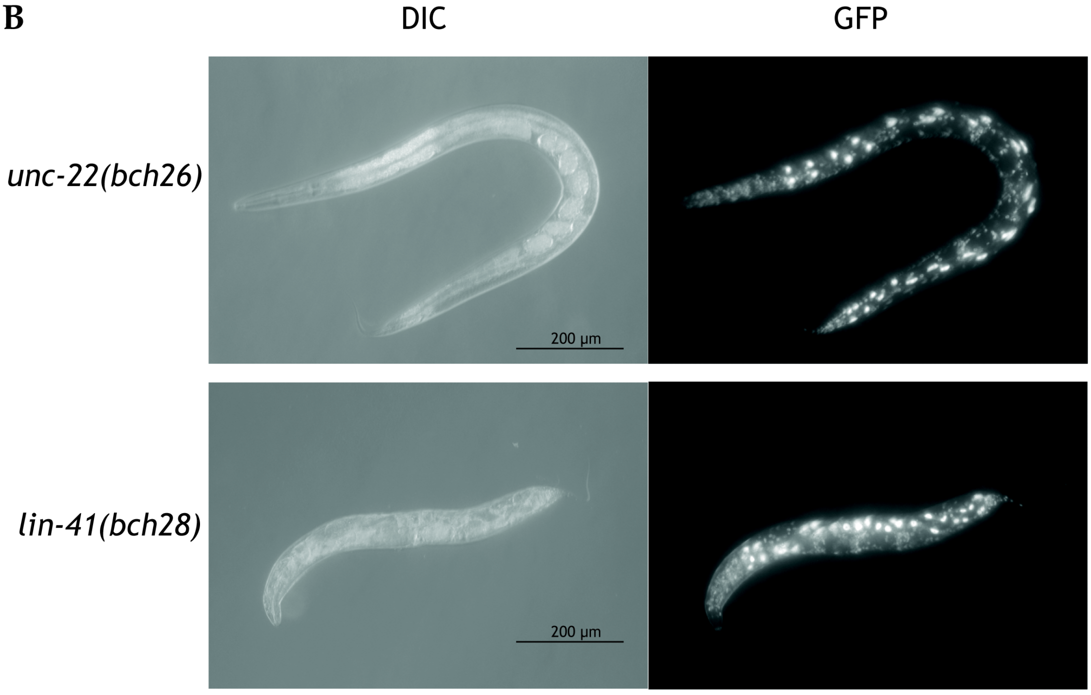

Figure S1. **Knock-in of a plasmid into a genomic locus upon non-homologous end joining-mediated repair of a Cas9/CRISPR lesion.**

A) PCR products spanning the junctions of *Peft-3::gfp::h2b::tbb-2 3'UTR* –containing plasmids into *unc-22* and *lin-41* loci. PCR primers are described in Materials and Methods. The arrow shows the oIK796-777 PCR product from *lin-41(bch28)* genomic lysates that was sequenced.
B) GFP expression from the *Peft-3::gfp::h2b::tbb-2 3'UTR*-containing plasmids in the *unc-22(bch26)* and *lin-41(bch28)* mutants.
