# Supplementary material for: CRISPR/Cas9 Genome Editing in Caenorhabditis elegans: Evaluation of Templates for Homology-Mediated Repair and Knock-Ins by Homology-Independent DNA Repair
Source: G3 (Bethesda). 2015 Jun 3;5(8):1649–56. doi: 10.1534/g3.115.019273 (PMC4528321; doi:10.1534/g3.115.019273)
Supplement: Supporting Information [file supp_g3.115.019273_FigureS2.pdf]

Figure S2.

a) Sequence of the 20th exon of the *unc-22* locus in N2

genomic sgRNA site (**PAM** bold)

AACCGCCGATGAAGTACAGTTTCCTGAACCCGTTGCCGAATACACAGGAGATCTACCGTACT
AAGCAAGCAGTGCTCACATGTAAAGTGAACACACCACGTGCTCCATTGGTATGGTACCG**TGG**
AAGCAAGGCTATTCAAGAAGGAGATCCACGATTCATTATTGAAAAGGATGCCGTCGGTCGTT
GTACACTTACAATCAAGGAAGTTGAGGAAGACGATCAAGCTGAATGGACTGCTAGAATCACA
CAAGACGTGTTCTCAAAGGTTCAAGTGTACGTTGAGGAGCCACGGCATACATTCGTTGTTCC
AATGAAGTCGCAAAAAGTCAACGAAAGTGATTTGGCAACATTGGAGACTGATGTTAACGACA
AGGATGCTGAAGTTGTTTGGTGGCATGATGGAAAGAGAATCGATATTGATGGAGTGAAATTC
AAGGTTGAATCTTCAAACAGAAAGAGAAGACTTATTATCAATGGAGCTAGAATTGAAGATCA
TGGAGAGTATAAGTGTACAACTAAGGATGATAGAACTATGGCTCAGCTCATCGTTGATGCTA
AGAATAAGTTCATCGTTGCTCTCAAAGACACTGAAGTTATTGAGAAGGATGATGTTACATTG
ATGTGTCAGACAAAGGACACAAAAACTCCTGGAATTTGGTTCCGTAATGGAAAACAAATTTC
CAGTATGCCCGGAGGAAAGTTCGAAACTCAATCGAGAAACGGAACTCATACTCTTAAAATCG
GAAAGATCGAGATGAACGAGGCTGATGTTTATGAAATCGATCAGGCAGGACTACGTGGATCT
TGCAATGTGACTGTTCTCGAGGCAGAAAAGCGTCCAATTCTCAACTGGAAGCCAAAGAAAAT
CGAAGCAAAGGCTGGAGAACCATGTGTTGTGAAGGTTCCATTCCAAATCAAGGGAACACGAC
GTGGAGATCCAAAGGCTCAAATTCTGAAGAATGGAAAGCCAATCGATGAAGAAATGAGAAAG
CTAGTTGAAGTTATTATCAAGGATGATGTGGCTGAGATTGTTTTCAAAAATCCACAACTTGC
TGATACAGGAAAGTGGGCTCTCGAACTCGGAAACTCGGCTGGAACAGCACTTGCTCCATTCG
AGTTGTTCGTTAAGGACAAGCCGAAACCACCAAAGGGTCCACTTGAAACCAAGAATGTTACT
GCTGAAGGTCTTGATCTCGTCTGGGGAACTCCAGATCCAGATGAGGGAGCTCCAGTTAAAGC
ATACATCATTGAAATGCAAGAGGGAAGAAGTGGAAACTGGGCTAAAGTTGGAGAGACTAAGG
GAACAGACTTCAAGGTTAAGGATCTTAAAGAACATGGAGAATACAAGTTCAGAGTCAAGGCT
CTTAATGAATGCGGACTCTCTGATCCACTCACAGGAGAATCTGTTCTTGCCAAAAATCCATA
CGGCGTTCCTGGAAAACCAAAGAACATGGACGCAATTGATGTTGACAAGGATCACTGTACCC
TTGCATGGGAACCGCCAGAGGAGGATGGAGGTGCTCCAATCACTGGTTACATCATTGAAAGA
AGAGAGAAGTCCGAGAAAGATTGGCATCAAGTTGGACAGACCAAACCAGATTGTTGTGAACT
GACTGATAAGAAGGTTGTCGAAGATAAGGAATACTTGTACAGAGTAAAAGCAGTCAACAAGG
CTGGACCAGGAGACCCATGTGATCATGGAAAGCCAATCAAGATGAAAGCCAAGAAAGCTTCT
CCAGAATTCACTGGTGGAGGCATCAAGGATCTTCGTCTTAAGGTCGGAGAAACTATCAAGTA
CGACGTTCCAATTTCTGGAGAACCACTCCCAGAATGTCTTTGGGTGGTTAATGGAAAACCAC
TGAAGGCTGTTGGAAGAGTCAAGATGTCTTCTGAAAGAGGAAAGCATATCATGAAGATCGAA
AATGCAGTTCGTGCTGATTCCGGAAAGTTCACTATCACTTTGAAGAACTCTTCTGGCTCATG
CGACTCGACCGCCACGGTCACTGTCGTTGGAAGACCAACTCCACCAAAGGGTCCACTCGATA
TTGCTGATGTTTGTGCCGATGGTGCAACCCTTTCCTGGAATCCTCCAGATGATGATGGAGGT
GATCCACTCACAGGATACATCGTTGAAGCTCAAGATATGGACAACAAGGGAAAATACATTGA
AGTTGGAAAGGTTGATCCAAACACCACTACCCTCAAAGTTAATGGACTCCGTAACAAGGGAA
ATTACAAGTTCCGCGTGAAGGCAGTCAACAACGAAGGAGAATCTGAGCCACTTTCTGCTGAT
CAGTACACTCAGATCAAGGATCCTTGGGATGAACCAGGAAAGCCTGGAAGACCAGAAATTAC
CGATTTCGATGCGGATAGAATTGACATTGCCTGGGAGCCACCACACAAAGATGGAGGAGCTC
CAATCGAGGAGTATATTGTCGAAGTTCGTGATCCAGATACCAAAGAATGGAAGGAAGTCAAG
AGAGTTCCAGACACCAATGCATCAATTTCTGGATTGAAGGAAGGAAAGGAATATCAGTTCAG
AGTTCGGGCTGTTAACAAGGCTGGGCCTGGACAACCTTCCGAACCATCAGAGAAGCAATTGG
CTAAGCCAAAATTCA

b) Sequence of the 20th exon of the *unc-22* locus in the *unc-22(bch26)* allele

remainder of the genomic sgRNA site
unknown
Peft-3 in forward orientation
remainder of the sgRNA site in the *Peft-3::gfp::h2b::tbb-2* 3'UTR plasmid
plasmid sequence

AACCGCCGATGAAGTACAGTTTCCTGAACCCGTTGCCGAATACACAGGAGATCTACCGTACT
AAGCAAGCAGTGCTCACATGTAAAGTGAACACACCACGTGCTCCATTGGTATGGGTCCTTTG
TATAGAAAAGGGTCCTTGTATAGAAAAGTTGGCACCTTTGGTCTTTTATTGTCAACTTCCA
TTGGTTCTTCCATTGTTTCTGTTAAATTAATGAATTTTTCATAAAATAAAGACATTATACAA
TATAAAAATGAAGAATTTATTGAAAATAAACTG[UNKNOWN SEQUENCE]
CAGATGCGTAAGGAGAAAATACCGCATCAGGCGCCATTCGCCATTCAGGCTGCGCAACTGTT
GGGAAGGGCGATCGGTGCGGGCCTCTTCGCTATTACGCCAGCTGGCGAAAGGGGGATGTGCT
GCAAGGCGATTAAGTTGGGTAACGCCAGGGTTTTCCCAGTCACGACGTTGTAAAACGACGGC
CAGTGAATTCGAGCTCGGTACCTCGCGAATGCATCTATAAG
GACAAGCCGAAACCACCGGAAGCAAGGCTATTCAAGAAGGAGATCCACGATTCATTATTGAA
AAGGATGCCGTCGGTCGTTGTACACTTACAATCAAGGAAGTTGAGGAAGACGATCAAGCTGA
ATGGACTGCTAGAATCACACAAGACGTGTTCTCAAAGGTTCAAGTGTACGTTGAGGAGCCAC
GGCATACATTCGTTGTTCCAATGAAGTCGCAAAAAGTCAACGAAAGTGATTTGGCAACATTG
GAGACTGATGTTAACGACAAGGATGCTGAAGTTGTTTGGTGGCATGATGGAAAGAGAATCGA
TATTGATGGAGTGAAATTCAAGGTTGAATCTTCAAACAGAAAGAGAAGACTTATTATCAATG
GAGCTAGAATTGAAGATCATGGAGAGTATAAGTGTACAACTAAGGATGATAGAACTATGGCT
CAGCTCATCGTTGATGCTAAGAATAAGTTCATCGTTGCTCTCAAAGACACTGAAGTTATTGA
GAAGGATGATGTTACATTGATGTGTCAGACAAAGGACACAAAAACTCCTGGAATTTGGTTCC
GTAATGGAAAACAAATTTCCAGTATGCCCGGAGGAAAGTTCGAAACTCAATCGAGAAACGGA
ACTCATACTCTTAAAATCGGAAAGATCGAGATGAACGAGGCTGATGTTTATGAAATCGATCA
GGCAGGACTACGTGGATCTTGCAATGTGACTGTTCTCGAGGCAGAAAAGCGTCCAATTCTCA
ACTGGAAGCCAAAGAAAATCGAAGCAAAGGCTGGAGAACCATGTGTTGTGAAGGTTCCATTC
CAAATCAAGGGAACACGACGTGGAGATCCAAAGGCTCAAATTCTGAAGAATGGAAAGCCAAT
CGATGAAGAAATGAGAAAGCTAGTTGAAGTTATTATCAAGGATGATGTGGCTGAGATTGTTT
TCAAAAATCCACAACTTGCTGATACAGGAAAGTGGGCTCTCGAACTCGGAAACTCGGCTGGA
ACAGCACTTGCTCCATTCGAGTTGTTCGTTAAGGACAAGCCGAAACCACCAAAGGGTCCACT
TGAAACCAAGAATGTTACTGCTGAAGGTCTTGATCTCGTCTGGGGAACTCCAGATCCAGATG
AGGGAGCTCCAGTTAAAGCATACATCATTGAAATGCAAGAGGGAAGAAGTGGAAACTGGGCT
AAAGTTGGAGAGACTAAGGGAACAGACTTCAAGGTTAAGGATCTTAAAGAACATGGAGAATA
CAAGTTCAGAGTCAAGGCTCTTAATGAATGCGGACTCTCTGATCCACTCACAGGAGAATCTG
TTCTTGCCAAAAATCCATACGGCGTTCCTGGAAAACCAAAGAACATGGACGCAATTGATGTT
GACAAGGATCACTGTACCCTTGCATGGAACCGCCAGAGGAGGATGGAGGTGCTCCAATCAC
TGGTTACATCATTGAAAGAAGAGAGAAGTCCGAGAAAGATTGGCATCAAGTTGGACAGACCA
AACCAGATTGTTGTGAACTGACTGATAAGAAGGTTGTCGAAGATAAGGAATACTTGTACAGA
GTAAAAGCAGTCAACAAGGCTGGACCAGGAGACCCATGTGATCATGGAAAGCCAATCAAGAT
GAAAGCCAAGAAAGCTTCTCCAGAATTCACTGGTGGAGGCATCAAGGATCTTCGTCTTAAGG
TCGGAGAAACTATCAAGTACGACGTTCCAATTTCTGGAGAACCACTCCCAGAATGTCTTTGG
GTGGTTAATGGAAAACCACTGAAGGCTGTTGGAAGAGTCAAGATGTCTTCTGAAAGAGGAAA
GCATATCATGAAGATCGAAAATGCAGTTCGTGCTGATTCCGGAAAGTTCACTATCACTTTGA
AGAACTCTTCTGGCTCATGCGACTCGACCGCCACGGTCACTGTCGTTGGAAGACCAACTCCA
CCAAAGGGTCCACTCGATATTGCTGATGTTTGTGCCGATGGTGCAACCCTTTCCTGGAATCC
TCCAGATGATGATGGAGGTGATCCACTCACAGGATACATCGTTGAAGCTCAAGATATGGACA

ACAAGGGAAAATACATTGAAGTTGGAAAGGTTGATCCAAACACCACTACCCTCAAAGTTAAT
GGACTCCGTAACAAGGGAAATTACAAGTTCCGCGTGAAGGCAGTCAACAACGAAGGAGAATC
TGAGCCACTTTCTGCTGATCAGTACACTCAGATCAAGGATCCTTGGGATGAACCAGGAAAGC
CTGGAAGACCAGAAATTACCGATTTCGATGCGGATAGAATTGACATTGCCTGGGAGCCACCA
CACAAAGATGGAGGAGCTCCAATCGAGGAGTATATTGTCGAAGTTCGTGATCCAGATACCAA
AGAATGGAAGGAAGTCAAGAGAGTTCCAGACACCAATGCATCAATTTCTGGATTGAAGGAAG
GAAAGGAATATCAGTTCAGAGTTCGGGCTGTTAACAAGGCTGGGCCTGGACAACCTTCCGAA
CCATCAGAGAAGCAATTGGCTAAGCCAAAATTCA
