# Supplementary material for: CRISPR/Cas9 Genome Editing in Caenorhabditis elegans: Evaluation of Templates for Homology-Mediated Repair and Knock-Ins by Homology-Independent DNA Repair
Source: G3 (Bethesda). 2015 Jun 3;5(8):1649–56. doi: 10.1534/g3.115.019273 (PMC4528321; doi:10.1534/g3.115.019273)
Supplement: Supporting Information [file supp_g3.115.019273_FigureS3.pdf]

Figure S3.

a) Sequence of the *lin-41* locus in N2 (partial)

lin-41 coding sequence
genomic sgRNA site (**PAM** bold)

gccacggttggcgaacgggtaaaaaggaagagccgatcgcctcgttactcaaggaaaaggct
cgacgtcgctggaggagggaaaagttggtttttaggataaaaaccaacttaaagtacaaaa
agagccggaaacggcgggaatgcgacgttggaaacgatcgtgatcttgaataaaggtgagga
actttaaataacaaagcaaaatgcgaaaatatattaaaaattaaaaacgtcgaaaaaaaat
taataaaattttttattcgaaaaatttgacctaccacaaatttttgactacttaatttt
ttgtttttgtttttattttcgaatattaaattgaaagagcggcatttttctgattttccca
tcaaaaatccttgtcatcagcagccctcgggccattttctctcatttgttccattatttt
catataaaaagtgcactttttttgtgcttcactgactttttgttcgattaattgttccatt
cgttctgaaaagtcaaaaaattcataagtattctaattgtagagtcatcgtttgccctttcc
actgataattaatcaacctttcagacttggaaaaagtgaaATGGCGACCATCGTGCCATGC
TCATTGGAGAAAGAAGAAGGAGCACCATCAGGACCTCGTCGGCTTCAAACTGAGATCGACG**T**
GGACGCCAACGACAGCGGAAACGAGCTGTCGATGGGCGGAAGCAGCAGTGAAGgtaacactt
gaaaattgaaaaaaaagcttccaacgaaacaacttcagGTGACTCAATGTCCCACCATCGCG
GTGAGCATTCACCAAACCATCATCATCAGGATAATCATCTTGGCTCGGGACCACCACCACCA
CAGTTCACTGGCTCACTATTTGACACTCCACCGTCAATGATTCAGTCACCACAGCAGCAACC
ACAGTTCCAGTTCAACACTGGATTCGGACTCGGACTCCCGCAAGACTCCTTTCGGTGCTCAG
TCTGCTCCAAGAGCTCGACGATTGGAGTGCTGCCGTTCGTTTGTGCGCACAAgtggggtttc
aagacaaattatcaaaatgacaattattttagAACGTGCCAATCATGCTACCAGATGACAC
CGTCCTCGTACGATCGACGGGgtaaataaatatctcaactttctgtatgatttatgaatttt
agCTTGCAAGCTGTGTGGTGCCGTATCGACAGCCACCGCCAACTTTACATCGCAGATGTACC
TGTCTCCGACGCTTCCATCACCGCCACGTGGAGCTCTGATGTCGGATTGTTCGACACCAACG
ATGAATAATCACATTAATTgtatgttttaattctcaatttacaccgaagaataacccaaatt
ccagCATCAACTCCACTTCATCAACCTCGTGCATTCTCATTCTCACTCAGTGGAATGCCTGG
ATCACCGTCTCCAGTGATGGGTGCACGGATGCCATCGTCTGCTGGTGGGCTCATGATGCGTC
CAATTGGATTCCCCGACTCGGACAGCAGTTTGACATCATGGTCTCCATTGCAACAACCATCA
CAGCTTTCTATCAATAATCTGTCGAGCATCGGAGGACACCAGCAACAATCGCCGATGTTGAT
GCAAAATGTGTTTGATTCGTTGGCAGTGgtcagtttttatcaaatttcaaacagagaaaagt
acaattttgtggagtgtggagctataaacaaattcaatttcccgaatttatgatgttatata
ttatgtggtcgcaaatcgaaaaatggttcaatttacggtttcgccgtcaatacctaacgaga
cccaacttctggggtggacctcgattaaaaaatgagcatttttctacggccccataatatt
tcaaaaatcatttttttcgcaaaaaaaagttagcatttctctcttcccaagaaacatatttt
tctccctggcttcaataagttcaataagtctttttctaattttttttgctaaatgcgccct
cctgcgagctgaaattgcacctaattcctcgtattctttacagAACGATGATACTCCTGTAT
TCTCTCCACTCTCTCCAACGAATACTTCAATGCATATGCCACCATCATTGATGGCATCACCA
GATGTTCCAAAACATTCTGCAACCATAGCTCCACCACGTAATTCCATGTGCTCGACGCCACG
TCTTCAATTGGCAACACCAATGTCTTCACAATCACAGCAAACTTTTCCAATTCCTTCACCAC
TTGGATCTCAGCCACAACAACAACAGCCAATGGGTCCGATTCAATGTCAAGGATGCGAATCG
AAAATgttagttttcccctaaaacttcaaagataattaaattatatttaaattttcagATC
GTTCGCCTACTGTATGCAATGTCAGGAAGCTTTGTGTATTCATTGTGTTCAAGCTCATCAGC
GTGTTCGAGCCACCAAACAACATGCGTTCGTTGAGCTTCAGCAGTTGATGGCTACTTTGgtt
agtttttatcttatcgtgtgttactacacgctttttgcgtctcaacacgacaattatttttt
tggaaattcataaaaagtatgcgcctttaaaacgtactgtaattaaacttttccattgtttt
tcattaaatatttgtatatttatttatcgaaaacctaaaattaaaaattaaaaccataaaaa
atcgatgaaaattcacagctacaaaaatttgaaactacagtactctttaaaggcgcacacct
ttttgtagtttacaaaaattgacattttgagacatgataccgtagtttgggagcaaaaaata

atatatgtggtctatattaatatcaccttgatccgtttcaagattctccacgtgcttgatga
cgtgaatcgtttgtaatcaggaatcataaaaagagtacgcaataatcataaatcaatgactc
ctgtcaatcaagaaaaacacagaatcagtactttttggtgtattttccgttttaaatg
tcttatcagtaactgattgcaggattagaacacaaaaattggctgccgaccgttccactaca
ttagtaaaacttcttgaaaataacttttgaaaactatcaaggaaagtatttcagctcactt
ttagattttaaatgaaacttgtgtcagcagtttcacattttcaaacaaaaccaagttcca
aaaagctcccagagtgtttgcttacctccaaacaccataaacatcaccaaaagcttgcaaaa
atccacatttcgaagaaaacaatttactgaaagcagtttcagagctgaaatactttccttac
aagtttatagatttttctatccgaatatgtccacgtactaggaaattcccatttttcaacat
ccaggcgttcgtttcccttttcttctccttcaaccaccttttttcgccattattcacaagc
cagtcggagggccaatttagtgccgagagagagagaggtgtctgctcctcatccggtgctct
ttttgcactatagtattatttttgctcggcgaacacgtaattcctctaaaatctaaaagta
ttattttcagATGAGCCGTGCAGTTCAACCACAACAAGCACAGCAATACACTCAAAATGTTG
GAGGATCAGTTCGTCAAGCACTTGGATCAGTTGGAAGTGGAGATGgtatat

b)Sequence of the *lin-41* locus in the *lin-41(bch28)* allele (partial)

lin-41(bch28)

lin-41 coding sequence
remainder of the genomic sgRNA site
unknown
truncated GFP
tbb-2 3'UTR
Peft-3 in reverse orientation
sgRNA site (reverse and complement) in the *Peft-3::gfp::h2b::tbb-2* 3'UTR plasmid; one base is deleted between the nucleotides in red
plasmid sequence

gccacggttggcgaacgggtaaaaaggaagagccgatcgcctcgttactcaaggaaaaggct
cgacgtcgctggaggagggaaaagttggtttttaggataaaaaccaacttaaagtacaaaa
agagccggaaacggcgggaatgcgacgttggaaacgatcgtgatcttgaataaaggtgagga
acttttaaataacaaagcaaaatgcgaaaatatattaaaaattaaaaacgtcgaaaaaaaat
taataaaatttttttattcgaaaaatttttgacctaccacaaatttttttgactacttaatttt
ttgtttttgttttattttcgaatattaaattgaaagagcggcatttttctgattttccca
tcaaaaatccttgtcatcagcagccctcgggccatttttctctcatttttgttccattatttt
catataaaaagtgcactttttttgtgcttcactgactttttttgttcgattaattgttccatt
cgttctgaaaagtcaaaaaattcataagtattctaattgtagagtcatcgtttgccctttcc
actgataattaatcaaccttttcagacttggaaaaagtgaaATGGCGACCATCGTGCCATGC
TCATTGGAGAAAGAAGAAGGAGCACCATCAGGACCTCGTCGGgtgctcatcattggaacaag
accgtcgttgccaagccaaaggacggaaagaagagacgtcatgcccgcaaggaatcgtactc
cgtctacatctaccgtgttctcaagcaagttcacccagacaccggagtctcctccaaggcca
tgtctatcatgaactccttcgtcaacgatgtattcgaacgcatcgcttcggaagcttcccgt
cttgctcattacaacaaacgctcaacgatctcatcccgcgaaattcaaaccgctgtccgttt
gattctcccaggagaacttgccaagcacgccgtgtctgagggaaccaaggccgtcaccaagt
acacttccagcaagtaaacccagctttcttgtacaaagtgggataaatgcaaaatcctttca
agcattcccttcttctctatcactcttctttctttttgtcaaaaattctctcgctaattta
tttgctttttaatgttattattttatgacttttatagtcactgaaaagtttgcatctgag
tgaagtgaatgctatcaaaatgtgattctgtctgatgtactttcacaatctctcttcaattc

cattttgaagtgctttaaacccgaaaggttgagaaaaatgcgagcgctcaaatatttgtatt
gtgttcgttgagtgacccaacaaaaagaggaaactttattgtgccgccaagaaaaaagtctc
acaactatcggatcc[UNKNOWN
SEQUENCE]CTCTCGGCGGGAGTGTTGCATACTTTTTCTCTCTGGCAGTTTATTTTCAATAA
ATTCTTCATTTTTATATTGTATAATGTCTTTATTTTATGAAAAATTCATTAATTTAACAGAA
ACAATGGAAGAACCAATGGAAGTTGACAATAAAAGACCAAAGGTGCcaactttctatacaa
agatctagatgcattcgcgaggtaccgagctcg**CGTCCACGTGATCTCAGTTTGAAGCCGAC**
cactggccgtcgttttacaacgtcgtgactgggaaaaccctggcgttacccaacttaatcgc
cttgcagcacatccccctttcgccagctggcgtaatagcgaagaggcccgcaccgatcgccc
ttcccaacagttgcgcagcctgaatggcgaatggcgcctgatgcggtattttctccttacgc
atctgtgcggtatttcacaccgcatatggtgcactctcagtacaatctgctctgatgccgca
tagttaagccagccccgacacccgccaacacccgctgacgcgccctgacgggcttgtctgct
cccggcatccgcttacagacaagctgtgaccgtctccgggagctgcatgtgtcagaggtttt
caccgtcatcaccgaaacgcgcgagacgaaagggcctcgtgatacgcctatttttataggtt
aatgtcatgataataatggtttcttagacgtcaggtggcacttttcggggaaatgtgcgcgg
aacccctatttgtttatttttctaaatacattcaaatatgtatccgctcatgagacaataac
cctgataaatgcttcaataatattgaaaaaggaagagtatgagtattcaacatttccgtgtc
gcccttattcccttttttgcggcattttgccttcctgtttttgctcacccagaaacgctggt
gaaagtaaaagatgctgaagatcagttgggtgcacgagtgggttacatcgaactggatctca
acagcggtaagatccttgagagttttcgccccgaagaacgttttccaatgatgagcactttT
CGATGGGCGGAAGCAGCAGTGAAGgtaacacttgaaaattgaaaaaaaagcttccaacgaaa
caacttcagGTGACTCAATGTCCCACCATCGCGGTGAGCATTCACCAAACCATCATCATCAG
GATAATCATCTTGGCTCGGGACCACCACCACCACAGTTCACTGGCTCACTATTTGACACTCC
ACCGTCAATGATTCAGTCACCACAGCAGCAACCACAGTTCCAGTTCAACACTGGATTCGGAC
TCGGACTCCCGCAAGACTCCTTTCGGTGCTCAGTCTGCTCCAAGAGCTCGACGATTGGAGTG
CTGCCGTTCGTTTGTGCGCACAAgtggggtttcaagacaaattatcaaaatgacaattattt
ttagAACGTGCCAATCATGCTACCAGATGACACCGTCCTCGTACGATCGACGGGgtaaataa
atatctcaactttctgtatgatttatgaattttagCTTGCAAGCTGTGTGGTGCCGTATCGA
CAGCCACCGCCAACTTTACATCGCAGATGTACCTGTCTCCGACGCTTCCATCACCGCCACGT
GGAGCTCTGATGTCGGATTGTTCGACACCAACGATGAATAATCACATTAATTgtatgtttta
attctcaatttacaccgaagaataacccaaattccagCATCAACTCCACTTCATCAACCTCG
TGCATTCTCATTCTCACTCAGTGGAATGCCTGGATCACCGTCTCCAGTGATGGGTGCACGGA
TGCCATCGTCTGCTGGTGGGCTCATGATGCGTCCAATTGGATTCCCCGACTCGGACAGCAGT
TTGACATCATGGTCTCCATTGCAACAACCATCACAGCTTTCTATCAATAATCTGTCGAGCAT
CGGAGGACACCAGCAACAATCGCCGATGTTGATGCAAAATGTGTTTGATTCGTTGGCAGTGg
tcagtttttatcaaatttcaaacagagaaaagtacaattttgtggagtgtggagctataaac
aaattcaatttcccgaatttatgatgttatatattatgtggtcgcaaatcgaaaaatggttc
aatttacggtttcgccgtcaatacctaacgagacccaacttctgggggtggacctcgattaa
aaaatgagcattttctacggccccataatatttcaaaaatcatttttttcgcaaaaaaaag
ttagcatttctctcttcccaagaaacatatttttctccctggcttcaataagttcaataagt
ctttttctaatttttttttgctaaatgcgccctcctgcgagctgaaattgcacctaattcct
cgtattctttacagAACGATGATACTCCTGTATTCTCTCCACTCTCTCCAACGAATACTTCA
ATGCATATGCCACCATCATTGATGGCATCACCAGATGTTCCAAAACATTCTGCAACCATAGC
TCCACCACGTAATTCCATGTGCTCGACGCCACGTCTTCAATTGGCAACACCAATGTCTTCAC
AATCACAGCAAACTTTTCCAATTCCTTCACCACTTGGATCTCAGCCACAACAACAACAGCCA
ATGGGTCCGATTCAATGTCAAGGATGCGAATCGAAAATgttagtttccccctaaaacttcaa
agataattaaattatatttaaattttcagATCGTTCGCCTACTGTATGCAATGTCAGGAAG
CTTTGTGTATTCATTGTGTTCAAGCTCATCAGCGTGTTCGAGCCACCAAACAACATGCGTTC
GTTGAGCTTCAGCAGTTGATGGCTACTTTGgttagtttttatcttatcgtgtgttactacac
gctttttgcgtctcaacacgacaattatttttttggaaattcataaaaagtatgcgccttta
aaacgtactgtaattaaacttttccattgtttttcattaaatatttgtatatttatttatcg
aaaacctaaaattaaaaattaaaaccataaaaaatcgatgaaaattcacagctacaaaaatt

tgaaactacagtactctttaaaggcgcacacctttttgtagtttacaaaaattgacattttg
agacatgataccgtagtttgggagcaaaaaataatatatgtggtctatattaatatcacctt
gatccgtttcaagattctccacgtgcttgatgacgtgaatcgtttgtaatcaggaatcataa
aaagagtacgcaataatcataaatcaatgactcctgtcaatcaagaaaaaacacagaatcag
tactttttggtgtatttttccgtttttaaatgtcttatcagtaactgattgcaggattaga
acacaaaaattggctgccgaccgttccactacattagtaaaacttcttgaaaataacttttt
gaaaactatcaaggaaagtatttcagctcacttttagatttttaaatgaaacttgtgtcagc
agtttcacatttttcaaacaaaaccaagttccaaaaagctcccagagtgtttgcttacctcc
aaacaccataaacatcaccaaaagcttgcaaaaatccacatttcgaagaaaacaatttactg
aaagcagtttcagagctgaaatactttccttacaagtttatagatttttctatccgaatatg
tccacgtactaggaaattcccatttttcaacatccaggcgttcgtttcccttttttcttctcc
ttcaaccacctttttttcgccattattcacaagccagtcggagggccaatttagtgccgagag
agagagaggtgtctgctcctcatccggtgctctttttgcactatagtattattttttgctcg
gcgaacacgtaattcctctaaaatctaaaagtattattttcagATGAGCCGTGCAGTTCAAC
CACAACAAGCACAGCAATACACTCAAAATGTTGGAGGATCAGTTCGTCAAGCACTTGGATCA
GTTGGAAGTGGAGATGgtatat
